# Sustainable bioactivity enhancement of ZnO–Ag nanoparticles in antimicrobial, antibiofilm, lung cancer, and photocatalytic applications

**DOI:** 10.1039/d3ra03736c

**Published:** 2023-09-06

**Authors:** Gouranga Dutta, Santosh kumar Chinnaiyan, Abimanyu Sugumaran, Damodharan Narayanasamy

**Affiliations:** a Department of Pharmaceutics, SRM College of Pharmacy, SRM Institute of Science and Technology Kattankulathur 603203 Tamil Nadu India damodhan@srmist.edu.in; b Department of Pharmaceutics, Faculty of Pharmacy, Karpagam Academy of Higher Education Eachaanari Coimbatore 641021 Tamilnadu India; c Department of Pharmaceutical Sciences, Assam University (A Central University) Silchar 788 011 Assam India abimanyu.s@aus.ac.in

## Abstract

Cancer, microbial infections, and water pollution are significant challenges the modern human population faces. Traditional treatments for cancer and infections often have adverse effects and ecological consequences, while chemical methods for water decontamination can produce harmful byproducts. Metal nanoparticles, particularly zinc oxide (ZnO) and silver (Ag) nanoparticles, show promise in addressing these issues. However, doping Ag on ZnO NPs may synergistically enhance biomedical and therapeutic effects with fewer adverse consequences and improved photocatalytic properties for wastewater treatment. This study aimed to create ZnO and ZnO–Ag nanoparticles through green synthesis and compare their anticancer, antimicrobial, and photocatalytic activity mechanisms. XRD studies determined the crystal diameters of ZnO NPs and ZnO–Ag NPs to be 12.8 nm and 15.7 nm, respectively, with a hexagonal wurtzite structure. The XPS and EDS analyses confirmed the presence of Ag on the ZnO NPs. ZnO NPs and ZnO–Ag NPs exhibited low aggregation in aqueous suspensions, with zeta potentials of −20.5 mV and −22.7 mV, respectively. Evaluating antimicrobial and antibiofilm activity demonstrates that ZnO–Ag NPs have superior potential to ZnO NPs and standard antibiotic drugs against *E. coli*, *S. typhi*, *B. subtilis*, *S. aureus*, *C. albicans*, and *A. niger*. The results of the *in vitro* cytotoxicity test indicated that on the NCI-H460 lung cancer cell line, ZnO NPs and ZnO–Ag NPs demonstrated IC_50_ values of 40 μg mL^−1^ and 30 μg mL^−1^, respectively. The photocatalytic degradation of methylene blue under direct sunlight revealed that ZnO and ZnO–Ag NPs degraded MB by 98% and 70% in 105 min, respectively. These results show that these nanomaterials may have great potential for treating the aforementioned issues.

## Abstract 2

Green synthesized ZnO and ZnO–Ag NPs using a plant extract. SEM image validate the final product. Characterized by FTIR, XRD, zeta, XPS, *etc.* Following this, their anticancer, antibacterial, and photocatalytic potentials are evaluated.

## Graphical abstract

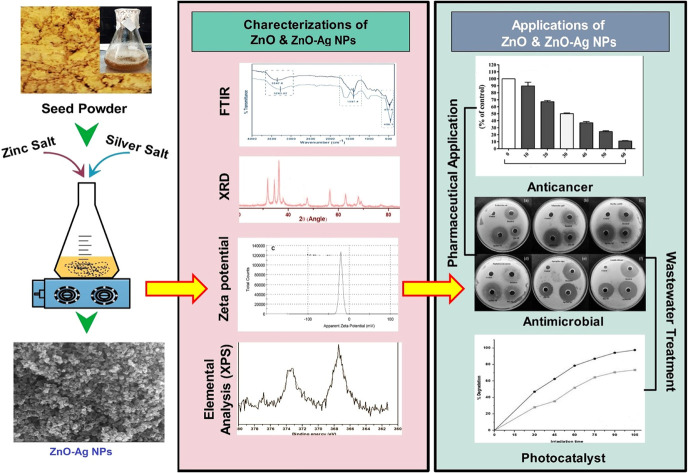

## 1. Introduction

The modern human population faces many significant challenges, including cancer, bacterial or fungal infections, and water pollution. Cancer is a multifaceted disease characterized by the organism's unrestrained proliferation and dissemination of abnormal cells. It represents a prominent contributor to mortality on a global scale, with a significant number of individuals receiving diagnoses annually. Cancer treatment typically encompasses surgical intervention, chemotherapy, and radiation therapy, which may incur substantial expenses, require significant time commitment, and result in notable adverse effects.^1,2^ In contrast, microbial infections arise from infiltrating pathogenic microorganisms, including bacteria, viruses, and fungi. These conditions can potentially induce various diseases, varying in intensity from minor to critical, and may pose a mortal danger if not properly addressed.^3,4^ The issue of water pollution has emerged as a crucial concern that has significant implications for both human health and the environment. Water pollution results from introducing harmful substances, including dyes, organic chemicals, and pathogens, into bodies of water, such as rivers, lakes, and oceans. The consequences of pollution can be severe, encompassing the transmission of diseases transmitted by water, the devastation of aquatic ecosystems, and the pollution of food supplies.^5–7^ Each of these issues necessitates distinct interventions, frequently entailing significant ecological consequences. Chemotherapeutic and antibiotic agents are commonly employed in managing cancer and bacterial infections; however, their synthesis and utilization may have substantial environmental effects.^8^ Numerous chemicals, including cationic and anionic dyes, such as methylene blue, methyl orange, eosin yellow, congo red, and bromophenol blue, as well as various other organic compounds, are discharged into both small and large water bodies by industries, such as printing, leather, paint, textile, and pharmaceuticals.^9^ These substances detrimentally affect the aquatic environment, posing risks to the health of animals and humans.^10^ The reaction between organic compounds in water and disinfectants can lead to harmful byproducts.^9,11^ The correlation between exposures to byproducts may increase susceptibility to cancer and biological issues in humans, and detrimental effects on aquatic organisms have been established.

The discovery of a substance that can efficiently address these concerns represents a significant advancement. In addition to simplifying treatment procedures and decreasing expenses, implementing this measure yields a more substantial beneficial effect on the ecosystem. Nanomaterials are a viable solution for simplifying and addressing these issues. Nanomaterials and nanoparticles have demonstrated significant advantages in the field of biomedical applications, particularly as carriers for delivering therapeutic agents. These tiny structures can be precisely engineered to specifically target desired cells or organisms, making them extremely valuable for various biomedical and therapeutic purposes.^12^ However, among the various types of nanomaterials, those composed of inorganic metals exhibit remarkable potential across diverse fields. Metal nanoparticles hold promise in the treatment of cancer and microbial infections, as well as in the decontamination of water bodies by eliminating pollutants and pathogens. Additionally, they offer the potential to reduce reliance on various chemotherapeutic and antibiotic drugs.^13,14^ Metal nanoparticles have been found to have practical applications in various fields, including optics, sensors, photocatalysis, semiconductors, pharmaceuticals, and biomedical applications. Owing to their unique attributes, such as a notable bandgap, optical characteristics, expansive surface area, and crystalline structures, that promote the scattering of subatomic particles, they exhibit adaptability for diverse applications.^15,16^

Nanoparticles of metal have demonstrated enormous potential in pharmaceutical and therapeutic applications, especially in treating microbial infections, malignancies, inflammations, and diabetes. The FDA has acknowledged the safety of zinc oxide nanoparticles (ZnO NPs) because of their safety and adaptability.^17^ They are suitable for biomedical applications owing to their antimicrobial, antitumor, antioxidant, tissue-repair, and drug-delivery properties.^13^ In addition to being effective photocatalysts, ZnO NPs can degrade hazardous industrial wastewater dyes.^18^ Similar to ZnO NPs, silver nanoparticles (Ag NPs) have similar properties and can be combined with zinc oxide nanoparticles (ZnO NPs) to enhance their biomedical and therapeutic effects. Bimetallic nanoparticles offer enhanced antimicrobial activity and high photocatalytic properties for eco-friendly wastewater purification.^19,20^ These metallic nanoparticles can be created through various methods. To synthesize these nanomaterials in an environmentally favorable way, green synthesis is the most sustainable approach, limiting chemical toxicity, high costs, and energy consumption. Plants contain numerous genetic variants that produce phytomolecules and compounds (vitamins, proteins, coenzymes, flavonoids, quinones, phenols, and carbohydrates) with functional molecules (–OH, –COOH, and –NH_2_) that can interact with metal ions, shrinking them to the nanoscale, stabilizing them as nanoparticles, and overcoming the limitations of conventional chemical synthesis.^13,21^

Our study offers a compelling analysis of ZnO and ZnO–Ag NP synthesis through an innovative green synthesis technique. We introduce the unexplored potential of *Trichosanthes dioica* seed extract as a biocatalyst for the preparation of metal nanoparticles. The present extract is sourced from the pointed gourd, indigenous to the Indian subcontinent. It comprises bioactive compounds that facilitate the production of ZnO and ZnO–Ag nanoparticles. To characterize the nanoparticles, various techniques were utilized: X-ray diffraction (XRD), scanning electron microscopy (SEM), transmission electron microscopy (TEM), X-ray photoelectron spectroscopy (XPS), and dynamic light scattering (DLS). The principal aim of our investigation is to fabricate nanoparticles of ZnO and ZnO–Ag and evaluate their multifaceted characteristics, encompassing their capacity as agents for combating cancer, inhibiting microbial growth, and resisting biofilm production and photocatalytic reactions. This study aims to broaden the current understanding of the biomedical applications of ZnO and ZnO–Ag NPs by utilizing a new combination of plant extract and a distinct cancer cell line. This approach tends to provide novel insights and contributes to the existing knowledge base. The results of our study reveal that the hybrid bimetallic composite exhibits superior performance compared to pure metal oxide nanoparticles. This finding has significant implications for treating cancer, microbial infections, biofilm inhibition and wastewater purification. In addition, our study contributes to the expanding body of knowledge in this area and paves the way for novel therapeutic interventions.

## 2. Experimental

### 2.1. Materials

The fruits of *Trichosanthes dioica* were acquired from local farmers in South 24 Pargana (West Bengal, India). Siscon Laboratories Pvt. Ltd (Tamil Nadu, India) supplied silver nitrate (AgNO_3_), zinc nitrate hexahydrate (Zn(NO_3_)_2_·6H_2_O), ethanol, sodium hydroxide (NaOH), dimethyl sulfoxide (DMSO), and methylene blue (MB). MTT (dimethyl thiazolyl tetrazolium bromide), Dulbecco's modified Eagle's medium (DMEM), phosphate buffer saline (PBS), fetal bovine serum (FBS), Mueller Hinton agar (MHA), and Sabouraud dextrose agar (SDA) were purchased from Hi-Media (Maharashtra, India). The chemicals acridine orange (AO), ethidium bromide (EB), and Hoechst dye were bought from Sigma-Aldrich (Tamilnadu, India). A human lung cancer (NCI-H460) cell line was acquired from the NCCS “(National Centre for Cell Science, Pune, India)”. All of the reagents and solvents are of analytical quality and are used immediately. The entire experiment was conducted using Milli-Q water (Millipore Sigma Aldrich).

### 2.2. Preparation of *Trichosanthes dioica* seed extract

*Trichosanthes dioica* seeds were collected from the fruits and washed under running tap water. The seeds were subjected to a week-long sun-drying process, after which they were finely pulverized to a mesh size of 80. A quantity of 5 g of seed powder was mixed with 100 mL of Milli-Q water and underwent boiling for 180 min at a temperature of 80 °C while being stirred magnetically. A pale yellow-colored extract was produced and passed through Whitman's filter paper. It was then stored in a refrigerator at a temperature of 4 °C for future experiments.

### 2.3. Synthesis of ZnO nanoparticles

A solution was made by mixing 0.2 M of zinc nitrate hexahydrate (Zn(NO_3_)_2_·6H_2_O) with 20 mL of Milli-Q water. Then, 20 mL of seed extract was placed on a magnetic stirrer and heated to 60 °C for 30 min before being added to the solution. The zinc nitrate solution was added into the extract solution dropwise at a continuous speed (approx. 30 drops per min), and the addition of a 0.1 M solution of NaOH increased the pH of the solution mixture. The solution mixture was on a magnetic stirrer at 60 °C for 2 h with a rotation speed of 1000 RPM (mixture A). After completion of 2 h, settlement of white color precipitate appeared. The mixture solution was centrifuged (4000 RPM), and the supernatant was discarded. The precipitated residual samples were washed 4–5 times with EtOH and Milli-Q water mixture (2 : 3) using a bath sonicator and centrifuge. The resulting product was dried for 8 h at a temperature of 120 °C. After drying, the product was calcinated for 2 h at a temperature of 400 °C.

### 2.4. Synthesis of ZnO–Ag nanoparticles

The experiment began by pouring 20 mL of seed extract into a magnetic stirrer, adding 0.1 M AgNO_3_, and stirring the liquid for 30 min at 60 °C. After 30 min, the resultant mixture was gradually added to “mixture A”, (which was constantly stirred at 1000 RPM for 2 h), at roughly 30 drops per min. The dropwise addition was performed, while “mixture A” was continuously stirred at the same speed. To maintain the basicity of the mixture, 0.1 M NaOH aqueous solution was slowly added and kept on the stirrer at 60 °C for 2 h, and a dark brown precipitate was collected. The product was dried at 120 °C for 8 h and calcinated for 2 h at a temperature of 400 °C.

### 2.5. Characterization of ZnO and ZnO–Ag nanoparticles

The green synthesized ZnO NPs and ZnO–Ag NPs have undergone a wide range of characterizations to understand their morphology, surface chemistry, size, *etc.* Using the aqueous medium, the absorption spectra were recorded at 200–800 nm using a UV-vis spectrophotometer (Shimadzu UV-3600, Japan). Fourier transform infrared spectrophotometry (SHIMADZU, Japan, IRTRACER 100) was used to analyze the surface chemistry and functionality of the metal oxide nanoparticles. The spectrum was recorded in the wavenumber range of 4000–400 cm^−1^. The crystalline nature of the ZnO and ZnO–Ag NPs was recorded using an X-ray diffractometer (BRUKER D8 Advance, USA), and scans were performed from (2*θ*) 10° to 90° at a rate of 5° min^−1^. The dynamic light scattering method was utilized to determine the hydrodynamic particle size and zeta potential (Malvern/Nano ZS-90, UK). Elemental analysis was determined from the XPS spectrum (PHI5000, USA). High-resolution SEM was carried out for the morphological evaluation of synthesized nanoparticles performed with Thermosceintific Apreo S, Scanning Electron Microscope. And the high-resolution TEM (JEOL Japan, JEM-2100 Plus transmission electron microscope). The elemental confirmation was evaluated using an EDAX (energy dispersive X-ray analyzer) equipped with the HRTEM.

### 2.6. Microbiological assay

#### 2.6.1. Test microorganisms

The antibacterial and antifungal properties of green synthesized ZnO and ZnO–Ag nanoparticles were evaluated against a range of harmful Gram-positive and Gram-negative bacteria as well as pathogenic fungi. The test organisms in the study were “*Escherichia coli* (ATCC 8739) and *Salmonella typhi* (MTCC 734) (Gram negative bacteria), *Bacillus subtilis* (NCIM 5433), and *Staphylococcus aureus* (ATCC 6538) Gram positive bacteria, and the fungi used for the antifungal study, *Aspergillus niger* (NCIM 1207) and *Candida albicans* (NCIM 3628)”.

#### 2.6.2. Zone of inhibition assay

An agar well-diffusion procedure was employed to measure the inhibitory zone and assess the antibacterial properties of the synthesized ZnO and ZnO–Ag nanoparticles against the aforementioned microorganisms. The process involved preparing solutions of MHA for bacteria and SDA for growing fungi. These solutions were sterilized using an autoclave (Osworld, JRIC-1, India) at a temperature of 121 °C and 15 psi for a duration of 15 min. After sterilization, the solutions were carefully poured into sterile glass Petri dishes using aseptic techniques. Four wells were made in each agar plate to hold microbial cultures with a concentration of 1 × 10^8^ CFU mL^−1^. Then, each well received 200 μL of the following: ZnO NPs (5 mg mL^−1^), ZnO–Ag NPs (5 mg mL^−1^), standard antibiotic drug Streptomycin/Oxytetaracyline (5 mg mL^−1^), and solvent control (DMSO 3% v/v). The microbial solution was evenly spread on the plates and allowed to disperse for 1 h. Subsequently, the plates were incubated at 37 ± 0.5 °C for 18–24 h for bacteria and 48–72 h for fungi. Then, measurements were acquired by determining the area around the well with inhibition of growth in millimeters. A greater zone of significant inhibition (>25 mm) against the examined microorganism was calculated.

#### 2.6.3. Estimating MIC and MBC levels for antimicrobial agents

A broth dilution technique was used to determine the minimum inhibitory concentration (MIC) of ZnO NP and ZnO–Ag NP. To perform an experiment, 100 μL of ZnO NP and ZnO–Ag NP solutions was diluted two-fold in a series of concentrations ranging from 1 to 1024 μg mL^−1^. Then, 100 μL of the diluted solutions was combined with 100 μL of a test culture containing 1 × 10^7^ CFU mL^−1^, along with 100 μL of MHA broth or SDA broth for bacteria or fungi, respectively, poured into 96-well plates. A 3% DMSO solution was used as a negative control. After incubation of the microorganisms in both wells, 30 μL of 0.05% resazurin was added to each well, which was then incubated for 2–4 h to observe a color change. Columns with no change in color after incubation had a high MIC value. The 100 μL of aliquots were taken from wells and inoculated onto Mueller Hinton agar and Sabouraud dextrose agar media to observe growth. The plates were then incubated under the previously stated conditions for bacteria and fungi. Afterward, they were investigated for the growth of microbial colonies. The lowest concentration of MBC/MFC indicated the absence of a viable microbial colony.^22^

#### 2.6.4. Anti-biofilm assay

ZnO NPs and ZnO–Ag NPs were investigated owing to their capacity to destroy the biofilms of different microorganisms using the Crystal Violet assay. To create the biofilms, a mixture of soya bean casein digest broth and 0.5% yeast extract was added to a cell suspension (1 mL) with a concentration of 1 × 10^7^ CFU mL^−1^ in a flat bottom 24-well plate, which was then incubated for 24 h. The wells were then filled with 100 μL of each nanoparticle and incubated under the previously mentioned conditions with ¼ Sub-MIC (sub-inhibitory concentrations) for tested microorganisms. Treated cells with 3% DMSO served as vehicle controls. The utilized medium was extracted and stained using 0.4% crystal violet on biological films. Following the dissolution of the biofilms in 95% ethanol, the absorbance was assessed using UV spectroscopy at a wavelength of 600 nm. Subsequently, the percentage of inhibition was determined based on the obtained measurements. Later, to observe the biofilm formation, both ZnO NPs and ZnO–Ag NPs were observed to suppress biofilm formation using optical microscopy. The biofilms were allowed to develop on 1 × 1 cm glass slides before being placed on 24-well titer plates. The wells were again prepared, and the samples were incubated for 24 h at 37 °C. The biofilm-coated glass slides were then air-dried and stained with crystal violet. A light trinocular microscope (Yamoto TM-100, Japan) was used at a magnification of 10× to observe the slides.^22^

### 2.7. *In vitro* cell culture assay

#### 2.7.1. Cell culture

The NCI-H460 cell line, which was derived from human lung large cell carcinoma, was obtained from NCCS in Pune. The cells were cultured in a controlled environment with a temperature of 37 °C, high humidity, and 5% CO_2_. The culture medium used was DMEM supplemented with 10% fetal bovine serum (FBS) and 1% streptomycin/penicillin.^23^

#### 2.7.2. Cytotoxic analysis

MTT assays were used to evaluate the cytotoxicity and cell viability of green-synthesized ZnO and ZnO–Ag NPs. Each well of a 96-well culture plate was seeded with about 1 × 10^4^ NCI-H460 monolayer cells and incubated overnight at 37 °C with 5% CO_2_. The cells were subsequently treated with nanoparticles at different concentrations (10–60 μg mL^−1^) for 24 h. A control culture was also set up and treated with serum-free DMEM. After 24 h, MTT solution was added, and the cultures were cultured for another 4 h. The formazan crystals were dissolved by adding DMSO, and the absorbance was measured using a microplate reader at 595 nm (measurement) and 620 nm (reference) (Bio-Rad, USA). Using the provided formula eqn (1), the proportion of inhibition was determined. Three times of each experiment were conducted, and the mean and standard deviation were determined.1
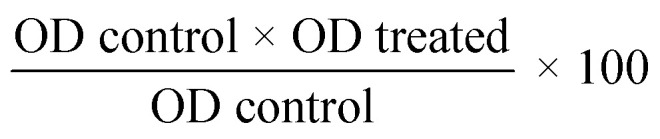
OD control = mean optical density of untreated cells; OD treated = mean optical density of treated cells.

#### 2.7.3. Apoptosis assay

The apoptosis assay of NCI-H460 cells against green-synthesized ZnO and ZnO–Ag NPs was identified using an AO and EtBr staining method [1 mg mL^−1^ for both AO and EtBr in 1 × PBS]. In a 6-well plate, 5 × 10^5^ NCI-H460 cells were seeded and allowed to attach overnight. After attachment, the cells were treated with the IC_50_ concentrations of ZnO and ZnO–Ag NPs in fresh media. Following 24 h of incubation, the cells were stained with AO/EtBr (10 μL) for 30 min, and excess staining was removed by washing with cold 1× PBS. The 6-well plate was mounted, and cell images were captured using an inverted fluorescent microscope (fluid cell imaging station).

#### 2.7.4. Reactive oxygen species generation assay (ROS)

To assess intracellular ROS, NCI-H460 cells were cultured and allowed to adhere overnight. After 24 h, the cells were incubated with serum-free DMEM media containing IC_50_ concentration of ZnO and ZnO–Ag nanoparticles and cultured for an additional 24 h. Next, cells were stained for 30 min with 40 μm DCFH-DA dye. 1× PBS was used to wash the stained cells. Photographs of the cells were obtained using a fluorescent microscope.

#### 2.7.5. DNA fragmentation assay by Hoechst (33258) staining

The Hoechst staining technique was used to determine cell DNA fragmentation. The cells were seeded and cultured overnight in a 6-well plate with a covered glass chamber. The half maximal inhibitory concentration (IC_50_) of ZnO and ZnO–Ag NPs was given to the cells, and they were kept in an incubator for 24 h. The cells were treated for 10 min with Hoechst stain. The cells were then rinsed with 1× PBS three times to remove excess dye. The images were captured using a fluorescence microscope (fluid imaging station).

### 2.8. Photocatalytic activity of dye degradation

The ability of ZnO and ZnO–Ag NPs to act as photocatalysts was determined by assessing their capacity to degrade methylene blue under exposure to solar light. The dye solutions were prepared (20 mg L^−1^) and scanned to determine the *λ*_max_ using a UV-Vis spectrometer. Later, 100 mL of methylene blue solution was mixed with 15 mg of ZnO NPs and ZnO–Ag nanoparticles. The mixed solution was sonicated for 10 min and magnetically stirred for 30 min under dark conditions to properly mix organic dyes and metal nanoparticles. After mixing, the solution was kept under direct sunlight. 5 mL of each sample was taken at 0, 30, 45, 60, 75, 90, and 105 min, and the nanoparticle was extracted using centrifugation (5000 RPM). The absorption of each sample was then measured using a UV-Vis spectrometer at a preset wavelength (*λ*_max_) to determine the degradation of the dyes. The % degradation efficiency was calculated using the following formula eqn (2):2
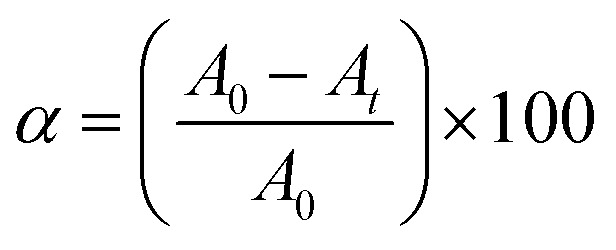
Here, *α* = dye degradation efficiency, *A*_0_ = absorbance at time 0, and *A*_*t*_ = absorbance at *t* time

### 2.9. Statistical analysis

The experimental data were analyzed using a one-way ANOVA with Graph Pad PRISM, USA. The experiment was repeated three times, with the findings represented as the mean value of the standard error. The statistical analysis revealed that the variances were statistically significant, with *p*-values less than 0.05.

## 3. Results and discussion

The green synthesis method is widely acknowledged as a prevalent alternative strategy for producing metal oxide nanoparticles. This approach entails the utilization of natural sources, such as extracts from plants, microorganisms, or biomolecules, which serve as agents that both reduce and stabilize. The utilization of the green synthesis method presents numerous benefits when compared to traditional synthesis methods.^13^ First, the utilization of non-toxic and sustainable materials is implemented, thus significantly contributing to the preservation of the environment. Additionally, it functions within moderate reaction conditions, thereby minimizing the requirement for harmful chemicals. Furthermore, this approach reduces reliance on costly machinery and lowers energy usage, leading to enhanced cost efficiency. Moreover, the utilization of diverse plant extracts or biomolecules results in the production of nanoparticles with a wide range of sizes and shapes.^24^ Metal oxide nanoparticles synthesized using green methods frequently demonstrate improved functional properties compared with nanoparticles synthesized using conventional methods. In addition, natural extracts or biomolecules contain organic compounds or functional groups that can provide supplementary therapeutic attributes and enhance their affinity with different chemicals or drugs, thereby enabling their effective delivery. This particular characteristic is may lacking in chemically synthesized nanomaterials.^13^

However, it is crucial to recognize the constraints linked to green synthesis techniques. The limitations encompass difficulties in achieving scalability owing to the inherent variations in natural extracts or biomolecules and the potential impact of batch-to-batch variations on reproducibility. Moreover, the existence of biomolecules can potentially result in stability concerns.^25^ To reduce these drawbacks, various approaches can be implemented to fabricate metal oxide nanoparticles for biomedical purposes. The green synthesized strategies require standardization and optimization to minimize variations between batches, applying purification techniques to enhance the purity and stability of nanoparticles. Surface functionalization to enhance the functionality of the surface, and implementing quality control and characterization measures to ensure the reliability and reproducibility of the results.^13,25^

**Fig. 1:**
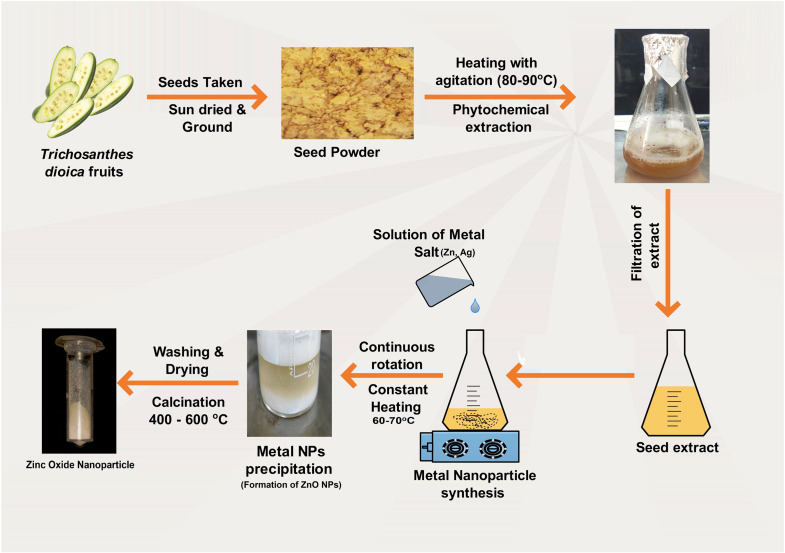
Schematic illustration of the green synthesis of ZnO and bimetallic ZnO–Ag NPs preparation.

In this study, ZnO NPs and ZnO–Ag hybrid bimetallic NPs were synthesized using a sun-dried seed extract of *Trichosanthes dioica* as a natural green precursor. This extract includes several phytochemicals that serve as reducing, stabilizing, and capping agents in the fabrication of nanoparticles. The development of nanoparticles has been identified visually as the mixture's color changed from yellow to pale white throughout the reaction, and the pale white precipitated, suggesting the formation of ZnO NPs. However, during the ZnO–Ag NP preparation reaction, the color changed from yellow to white to brown, indicating the formation of the ZnO–Ag nanoparticles. When the two nanoparticles are washed and dried, they turn into a fine powder (Fig. 1). This suggests that the numerous functional groups present in the phytochemicals of the seed extract account for nanoparticle transformation. Temperature serves a crucial function in the synthesis of ZnO–Ag nanoparticles. In this procedure, the temperature was primarily used twice. Temperature is a crucial factor in regulating reaction time, reaction rate, *etc.*, during the synthesis of metal NPs. Increasing temperatures can accelerate the reduction of metal compounds and facilitate the formation of nanoparticles. However, excessive heat can result in unintended reactions, such as particle aggregation and morphological changes. After the synthesis of metal NPs, they must be dried out and calcinated at extremely high temperatures, which reduces impurities, modifies the crystal structure and morphology, and creates stable nanoparticles. Owing to these factors, the optimal temperature is necessary for synthesizing ZnO–Ag NPs and calcining the product to produce stable nanoparticles.^26–28^ Many researchers have analyzed the physical properties and morphological differences resulting from the synthesis and calcination temperature changes. Kem *et al.* produced ZnO NPs using a green synthesis method and calcined the product at 600, 700, 800, and 900 °C for one hour at each temperature. They discovered that increasing the temperature can alter particle morphologies and physical and surface properties.^27^ In a similar study conducted by Álvarez-Chimal *et al.*, it was determined that changes in various physical properties determine the efficacy of metal NPs.^26^ We carefully selected the temperatures for the synthesis, drying, and calcination procedures based on a thorough literature review. Our preliminary trials ensure optimal conditions and achieve superior results.

**Fig. 2:**
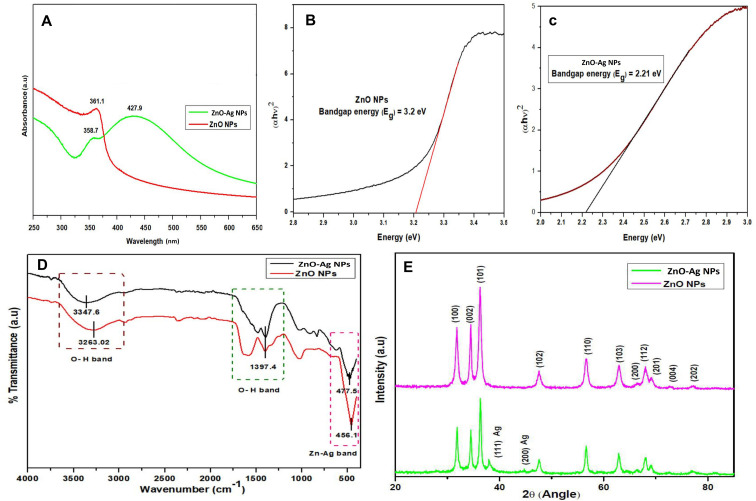
(A)–(C) The UV spectrum with Tauc plot of ZnO NPs and ZnO–Ag NPs, (D) FTIR spectrum of ZnO and ZnO–Ag NPs, and (E) XRD spectrum of ZnO and ZnO–Ag NPs.

A UV-Vis spectrometer was used to examine the optical characteristics of the ZnO and ZnO–Ag NPs produced by green synthesis. The analysis was conducted in a wavelength range of 200–800 nm, and the resulting data were presented as absorbance *vs.* wavelength (*λ*). The *λ*_max_ determined for the ZnO NPs was 361.1 nm although the ZnO–Ag NPs show a high intense *λ*_max_ at 427.9 nm in the visible light region and a low intense peak at 358.7 nm, representing the presence of ZnO NPs (Fig. 2A). The red shift towards the longer wavelength observed in the absorbance plot was due to the conjugation of the Ag over the ZnO NPs.^29,30^ The presence of surface plasmon resonance (SPR) in nanoparticles corresponds to the significance of this peak at 427.9 nm. SPR is a phenomenon in which oscillation in electrons on the surface of a metal (in this case, Ag) interacts with incident light, leading to enhanced absorption and scattering of light at particular wavelengths.^31,32^ The noticeable peak at 427.9 nm confirms the successful generation of SPR effects by indicating the presence of Ag nanoparticles on the surface of ZnO. This plasmonic behavior is responsible for the enhanced optical and photocatalytic properties observed in bimetallic ZnO–Ag nanoparticles. The visible light photocatalytic activity is increased owing to the SPR-mediated light absorption of Ag nanoparticles, which enhances electron transfer processes.^29^ Therefore, the significant peak at 427.9 nm in the visible light region represents SPR, emphasizing the presence of Ag and its essential role in improving the photocatalytic performance of bimetallic ZnO–Ag nanoparticles.^29,32^ The bandgap energy (*E*_g_) was also determined for both nanoparticles using Tauc's equation eqn (3):

3 (*αhν*)^*n*^ = *A*(*hν* − *E*_g_),

where *α* = absorption coefficient, *hν* = photon energy, *A* = constant, and *E*_g_ = optical bandgap.

The calculated bandgap of the ZnO NPs was found to be 3.2 eV (direct), while that of ZnO–Ag NPs was determined at 2.21 eV (direct). The bandgap reduction is due to the incorporation of silver on the ZnO NP surface, which is formed as hybrid ZnO–Ag NPs (Fig. 2B and C). This result indicates that in the presence of UV and visible light, ZnO–Ag NP can be used as a photocatalyst. Moreover, ZnO NPs can be used as photocatalysts under UV – *A*(315–400 nm) light.^33^

The FTIR spectra of ZnO and ZnO–Ag NPs were measured using the KBr pellet procedure in a scanning range of 4000–400 cm^−1^. Significant peaks at 3347.6 and 3263 cm^−1^ in both spectra show the bending vibration of the O–H band (hydroxyl group) caused by the presence of surface water molecules. The other distinctive peak in both spectra was seen at 1397.4 cm^−1^, representing the bending mode of the O–H band (Fig. 2B). Significant absorption peaks at 456.1 cm^−1^ and 477.5 cm^−1^ are due to the stretching vibration of ZnO and ZnO–Ag NPs, indicating the existence of metal nanoparticles (Zn–O).^34^ On the surface of the nanoparticles, the presence of the –OH functional group may reflect phytochemical residue or ambient moisture. The peak intensity is decreased in ZnO–Ag nanoparticles owing to the formation of Ag nanoparticles on the exterior of ZnO nanoparticles. This clearly validates the creation of silver nanoparticles on the surface of ZnO nanoparticles.^35,36^

The structural properties and purity of the crystals of ZnO NPs and ZnO–Ag nanoparticle were analyzed by performing XRD analysis. The peak positions obtained for ZnO NPs with 2*θ* values are 31.7°, 34.4°, 36.26°, 47.6°, 56.61°, 63.02°, 68.01°, 69.19° and 77.29° index the lattice planes of (100), (102), (101), (102), (110), (103), (112), (201) and (202), respectively. These peaks are attributed to the hexagonal wurtzite structure of green synthesized ZnO NPs. The XRD pattern was found to be similar to that of the Joint Committee on Powder Diffraction Standards (No. 36-1451).^36^ In ZnO–Ag NPs, the Ag showed two low-intensity peaks at the 2*θ* values of 37.94° and 44.76° with lattice planes of (111) and (200), respectively. The crystalline sizes of ZnO NPs and ZnO–Ag nanoparticles were determined to be 12.8 nm and 15.7 nm, respectively, by applying the following Debye–Scherer equation eqn (4) (Fig. 2C):^33,34^4
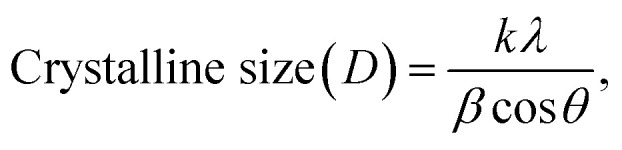
where *β* = FWHM, *θ* = Bragg angle, *k* = 0.9, and *λ* = 0.15409 (X-ray wavelength).

**Fig. 3:**
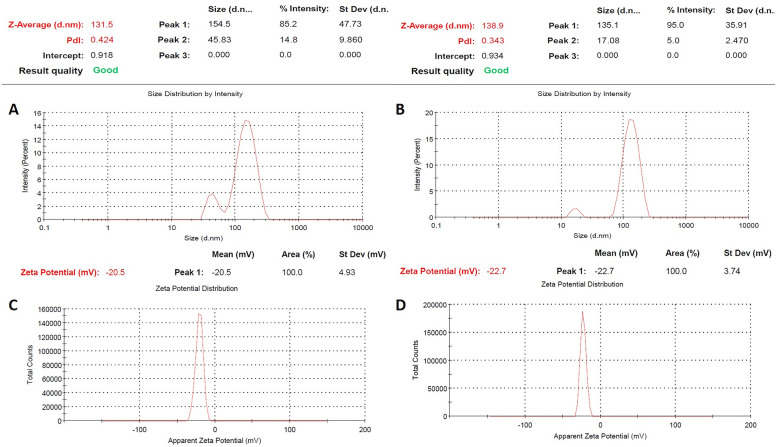
(A) and (B) Particle size distribution of ZnO and ZnO–Ag NPs using the DLS method. (C) and (D) Zeta potential spectrum of ZnO and ZnO–Ag NPs.

Dynamic light scattering (DLS) measurements were used to evaluate hydrodynamic particle size. Both calcinated samples were dispersed in Milli-Q water (1 mg 10 mL^−1^) by sonication. The average hydrodynamic particle size (*Z*-average) of ZnO NPs was determined to be 131 nm, whereas that of ZnO–Ag nanoparticles was 138 nm (Fig. 3A and B). The PDI (polydispersity index) was 0.424 and 0.349 for puro ZnO and ZnO–Ag NPs, respectively. These findings suggest that the dimensions of the crystals, as determined by XRD, are considerably lesser than the dimensions of the particles ascertained by DLS.^37^ In this study, the stability of the colloidal suspension of both nanoparticles was determined by continuously measuring the hydrodynamic particle size and zeta potential over four weeks. On the first day, data were collected by preparing the sample described above and storing it undisturbed at 4 °C. The subsequent measurements were taken after 1 week, 2 weeks, 3 weeks, and 4 weeks, as shown in Table 1. It was evident from the measured sizes that the particles in the aqueous suspension slightly increased in size from the first day (131 nm for ZnO and 138 nm for ZnO–Ag) to the second week (142 nm for ZnO and 153 nm for ZnO–Ag) and then to the end of the fourth week (261 nm for ZnO and 240 nm for ZnO–Ag). Moreover, we observe that during storage, both particulates tend to settle down to the bottom of the suspension and are readily dispersed by slightly jostling the storage container.

**Table 1:** Colloidal stability study of the green synthesized ZnO NPs and ZnO–Ag NPs

Time (weeks)	Zeta potential (mV)		Average hydrodynamic size (nm)	
	ZnO NPs	ZnO–Ag NPs	ZnO NPs	ZnO–Ag NPs
Initial day	−20.5	−22.7	131	138
1st week	−19.9	−21.8	142	153
2nd week	−19.3	−21	175	166
3rd week	−18.5	−20.4	227	183
4th week	−17.4	−19.1	261	240

The DLS determines the dimensions of nanoparticles within a dispersed medium by analyzing their diffusion characteristics, representing each particle's distinct physical properties. The behaviour of nanoparticles can be influenced by various interactions when distributed in a liquid medium. The aggregation phenomenon arises from the coalescence of nanoparticles, which may be facilitated by attractive forces, such as van der Waals forces or electrostatic interactions, leading to an increase in the hydrodynamic size of the resulting particles. These aggregated structures may exhibit greater prominence in aqueous dispersions, resulting in larger hydrodynamic sizes than the crystalline size determined through XRD.^37,38^ Complementary characterization techniques, such as TEM, were employed to enhance understanding of the particle size distribution and the existence of aggregates (Fig. 3A and B). The zeta potentials of ZnO and ZnO–Ag nanoparticles were measured and recorded as −20.5 mV and −22.7 mV, respectively, as depicted in Fig. 3C and D. The zeta potential measures the electrical potential difference between the surface of nanoparticles and the surrounding dispersion medium, indicating their stability and behaviour.^39^ The dissimilarity in the zeta potentials of ZnO and ZnO–Ag NPs, which were recorded as −20.5 mV and −22.7 mV, respectively, indicates that the introduction of silver to ZnO nanoparticles slightly affects the surface charge. Moreover, the acquired data suggest significant repulsion among the nanoparticles. The 4 weeks study found that both of the nanoparticle's zeta potential decrease in the colloidal suspension over time. This may be because of the particle's aggregation caused by various factors, such as absorption of ions, different electrostatic interactions, *etc.* This leads to changes in the surface charge of the nanoparticles. The surface charge affects the particle's hydrodynamic size in suspension form. If the potential decreases, the repulsion force between the nanoparticles lowers, leading to particle aggregation. In this study, it is clearly shown that during the period, the zeta potential of the particle decreases, which leads to increases in the average hydrodynamic size of the particle.^39,40^ Holišová *et al.* developed gold nanoparticles (NPs) using plants and assessed their stability over a period of five weeks by monitoring their zeta potential. The researchers observed a gradual decrease in the zeta potential of the colloidal suspension of Au NPs from the beginning of the experiment to the fifth week.^41^

**Fig. 4:**
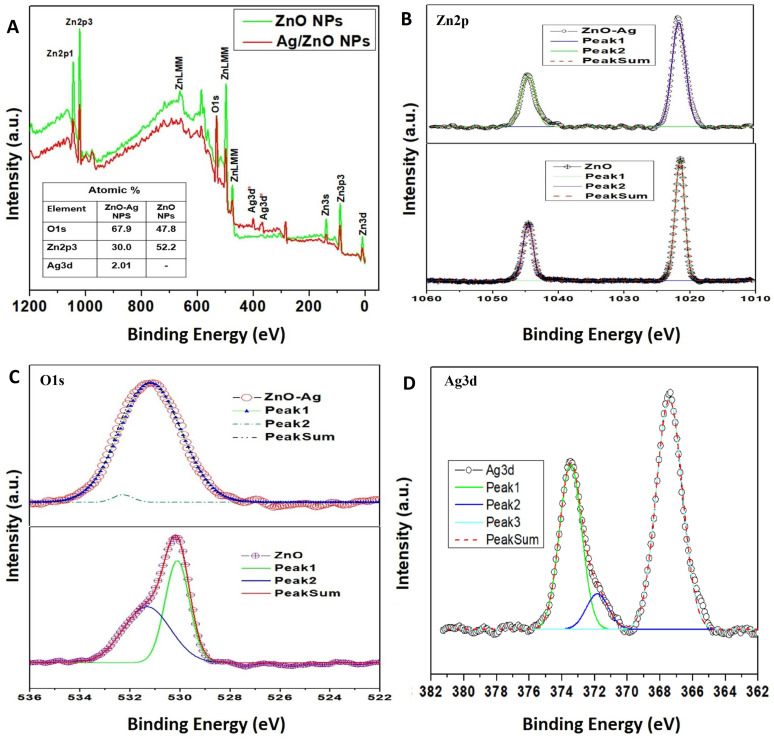
(A) The XPS spectrum of ZnO NPs and ZnO–Ag NPs; (B)–(D) individual high-resolution spectrum of Zn2p, O1s, and Ag3d, respectively.

The composition of the green-synthesized ZnO and ZnO–Ag NPs was confirmed by performing XPS analysis, which revealed the presence of Zn, O, C, and Ag in the examined material. This is depicted in Fig. 4. The XPS spectra of ZnO NPs and ZnO–Ag NPs are demonstrated in Fig. 4A. No other significant peak was observed, which provides evidence of the purity of the sample. In the high-resolution spectra of Zn shown in Fig. 4B, two nearly symmetrical peaks were observed at 1020.67 eV and 1043.47 eV for ZnO NPs, and at 1021.59 eV and 1044.15 eV for ZnO–Ag NPs. These peaks correspond to Zn2p^3/2^ and Zn2p^1/2^, respectively, confirming the presence of zinc in the oxidation state of Zn^2+^ in both compounds.^42,43^Fig. 4C displays the O1s spectra of ZnO and ZnO–Ag nanoparticles. Two Gaussian curves with maxima at 528.74 eV and 530.12 eV are fitted to the ZnO NP curve. This shows the presence of a surface hydroxyl group and chemically absorbed oxygen. One curve is produced at 531.24 eV for ZnO–Ag NPs, which corresponds to the oxygen in ZnO NPs.^44,45^Fig. 4D depicts XPS data with distinct peaks at 373.56 eV and 369.72 eV. These summits correspond to the Ag3d^3/2^ and Ag3d^5/2^ energy levels. These specific peaks strongly suggest that silver (Ag) is present in the sample. Observation of the Ag3d^3/2^ and Ag3d^5/2^ peaks provides conclusive evidence that silver is present on the sample's surface.^32,45^

**Fig. 5:**
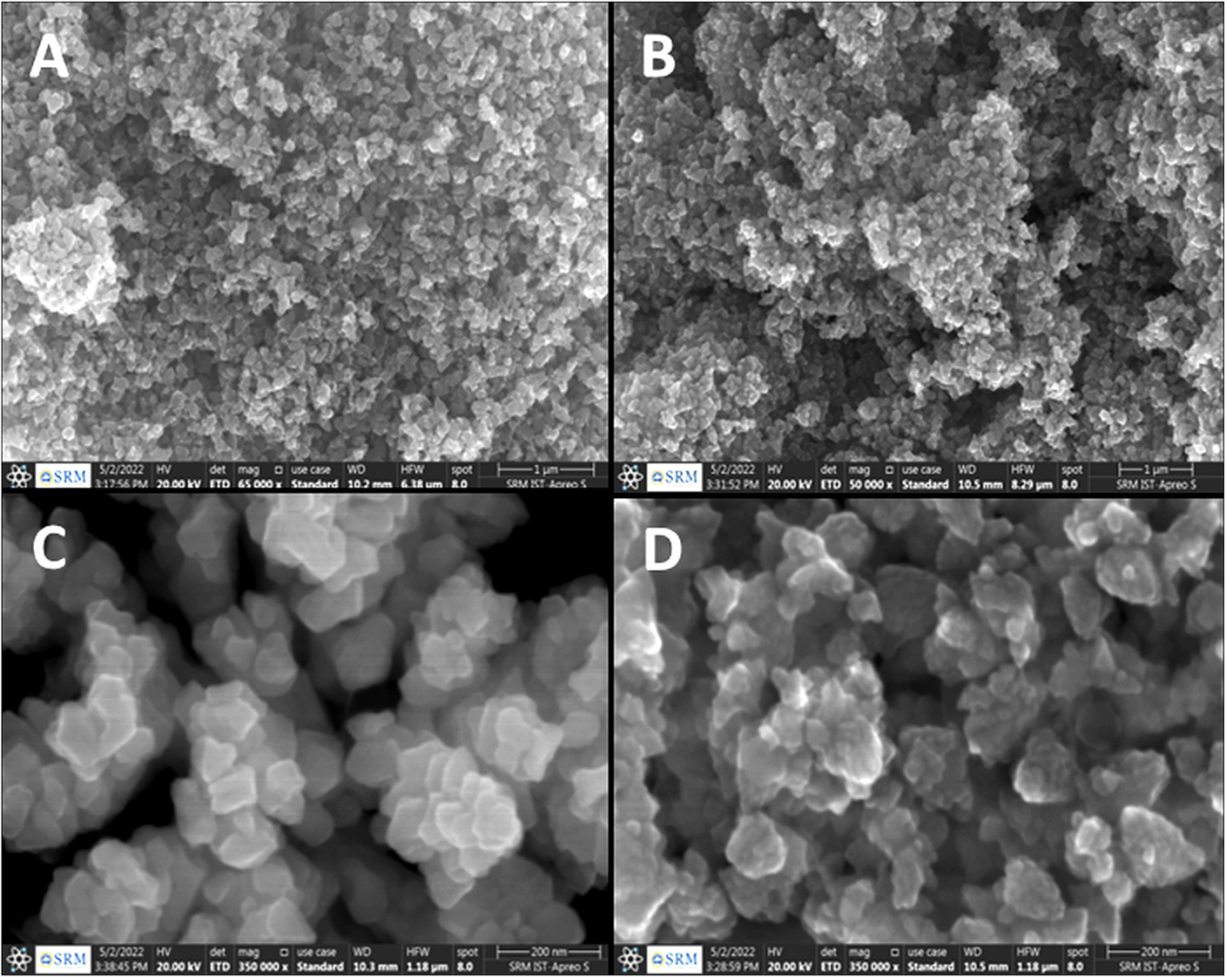
SEM images of (A) and (C) ZnO NPs; (B) and (D) ZnO–Ag NPs ((C) and (D) are higher resolutions of (A) and (B)).

The HRSEM examined the surface morphology of the sample at 20 kV acceleration voltages to determine the distribution and shape of the nanoparticles. Ten μL of the nanoparticle suspension were placed on aluminum foil and dried at 80 °C to evaporate the solvent from the thin layer. Different magnifications were used to capture images for both types of investigative materials. At lower magnification, the distribution of the two nanomaterials (ZnO and ZnO–Ag depicted in Fig. 5A and B, respectively) reveals an aggregated structure with rough edges. Higher magnification images reveal that the particles of both materials are roughly spherical and densely packed. For ZnO–Ag nanoparticles (Fig. 5D), the particle surfaces are rougher and slightly larger than those for ZnO NPs (Fig. 5C), which may suggest the development of silver on the ZnO NP surface. The particle size found by HRSEM is nearly in the range of 90–120 nm, which has proven the reduced aggregation of nanoparticles, as it is found to be higher than the determined crystalline size.^33,46^ For clear visualization, another microscopic technique, TEM, was employed.

**Fig. 6:**
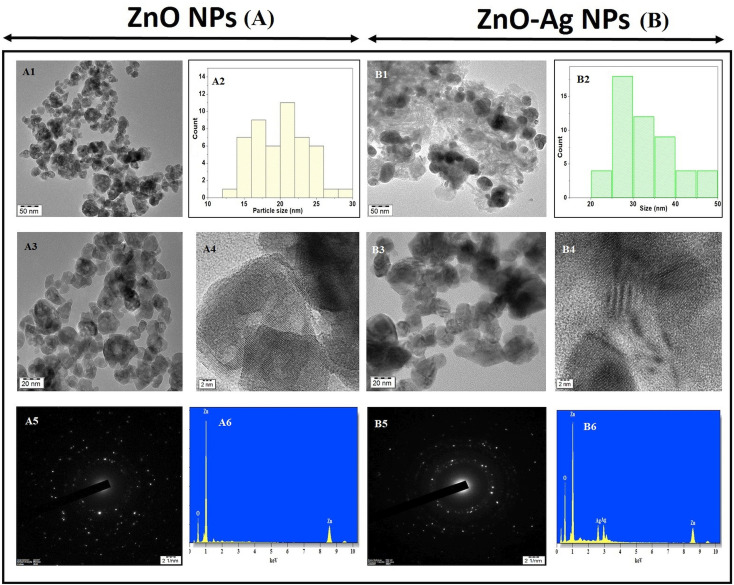
TEM, SAED images and bar diagram of particle size of ZnO NPs (A1–A5), ZnO–Ag NPs (B1–B5); EDAX spectrum of ZnO NPs (A6) and ZnO–Ag NPs (B6).

The utilization of high-resolution transmission electron microscopy (HRTEM) was implemented to conduct a thorough observation of the ZnO NPs and ZnO–Ag nanoparticles. The data presented comprised patterns obtained through the utilization of selected area electron diffraction (SAED). The images shown in Fig. 6A1, 6A2, and 6A4 illustrate the morphology of ZnO nanoparticles, exhibiting a roughly spherical shape and varying magnifications. As demonstrated, the ZnO–Ag nanoparticles are depicted at equivalent magnification in Fig. 6B1, 6B2, and 6B4. The presented data indicate that the particles predominantly exhibit spherical morphology. The dispersion of the formed nanoparticles is represented through bar diagrams in Fig. 6A2 and 6B2. The graphical representations illustrate that the ZnO nanoparticles exhibit a size distribution within the 15–25 nm range, with a mean diameter of 20.28 nm. Conversely, the ZnO–Ag nanoparticles display a size range of 20–50 nm, with an average diameter of 29.77 nm. The graphical representations, including the images and particle dispersion bar diagram, indicate that the measured particle sizes exceed the crystalline sizes (12.8 nm and 15.7 nm) that were ascertained through XRD and were much lower than the hydrodynamic size through DLS for both categories of nanoparticles. The variance in length is attributed to the amalgamation of crystallites during the making of the colloidal dispersion from the calcinated/dried nanoparticle. Observing intense regions in the selected area electron diffraction (SAED) ring patterns depicted in Fig. 6A5 and 6B5 provides evidence for producing nanoparticles with crystallinity. This finding is suggestive of the crystallographic planes of both ZnO and Ag. The elemental mapping process was executed using HRTEM with energy-dispersive X-ray spectroscopy (EDAX) attachment, as depicted in Fig. 6A6 and 6B6. The nanomaterials were investigated using HRTEM, and the resulting EDAX spectrum revealed the presence of zinc, oxygen, and silver, while no other material was detected.^47,48^ The impact of nanoparticle size is particularly significant when considering ZnO and ZnO–Ag NPs. It is widely recognized that as the size of nanoparticles decreases, there is an increase in the ratio of surface area to volume. This phenomenon leads to enhanced reactivity and catalytic activity. The efficacy of the nanoparticle can potentially be enhanced by altering its shape, thereby improving its surface area. Many studies have investigated the antibacterial and cytotoxic properties of ZnO nanoparticles of varying sizes (ranging from nanometer to micrometer) and shapes (including spherical, cubical, rod-like, flower-like, and hexagonal).^49–51^ Numerous studies have demonstrated that manipulating the size and shape of nanoparticles can effectively enhance the specific surface area of the particles, thereby exerting a notable impact on antibacterial efficacy against various microorganisms. Similarly, it has been observed that smaller nanoparticles (NPs) exhibit enhanced cellular uptake owing to their augmented surface area and enhanced ability to penetrate cell membranes. The heightened internalization process can amplify cytotoxicity because of the intensified interaction between nanoparticles (NPs) and cellular constituents.^50,52^

Babayevska *et al.* (2022) synthesized a series of zinc oxide nanoparticles (ZnO NPs) exhibiting diverse morphologies and size distributions from the nanometer to the micrometer scale. The results obtained from the N_2_ adsorption and desorption analysis indicate that ZnO nanoparticles and nanorods exhibit a greater specific surface area. Consequently, it was observed that nano-range particles exhibited the most potent antibacterial and cytotoxic effects.^50^ For example, González *et al.* generated three variations of ZnO nanoparticles, each exhibiting varying sizes and shapes. Upon assessing antibacterial and anticancer efficacy across various microorganisms and cell lines, it was determined that the smallest particle consistently exhibits the highest activity level in all tested applications.^53^ In a separate investigation, Talebian *et al.* conducted an experiment in which they synthesized three distinct variations of ZnO particles characterized by their respective morphologies: flower-like, hexagonal rod-like, and spherical-like structures. Recent research has revealed that ZnO particles exhibiting a flower-like morphology enhance antibacterial and photocatalytic properties compared to other ZnO particle configurations. The flower-shaped particle exhibits enhanced reactivity owing to its multiple pallets, contributing to an increased surface area.^49^ A shape-dependent experiment was conducted by Mohamed *et al.* to assess the potential of two distinct shapes of ZnO nanoparticles. It has been observed that nanorod-shaped ZnO nanoparticles exhibit superior performance across various applications compared to hexagonal-shaped nanoparticles. These examples clearly demonstrate that the size and shape of nanoparticles play a significant role in determining their properties and activity.^54^

Our research shows that the particles under investigation exhibit dimensions within the nanometer scale and possess a predominantly spherical morphology with greater applicability. Specifically, the particle sizes for ZnO and ZnO–Ag nanoparticles fall in the ranges of 15–25 nm and 20–50 nm, respectively. The antibacterial and anticancer effects were assessed, and the findings are presented herein.

**Fig. 7:**
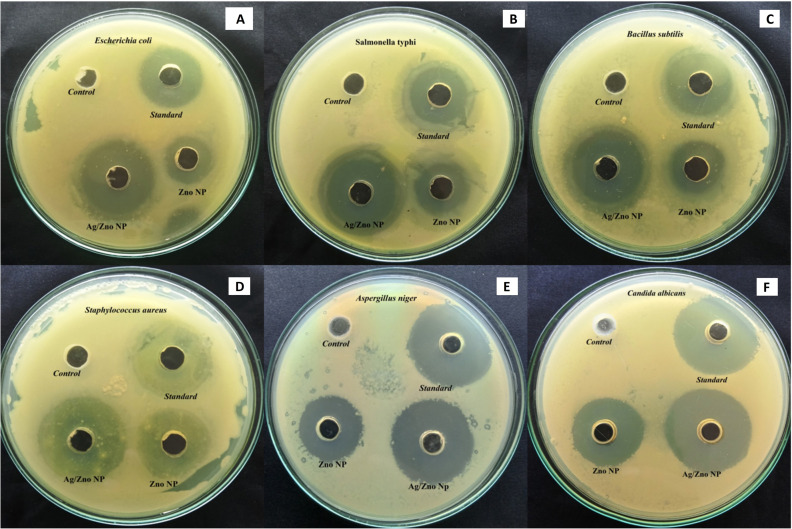
Agar well-diffusion assay of (A) *E. coli*, (B) *S. typhi*, (C) *B. subtilis*, (D) *S. aureus*, (E) *A. niger*, (F) *C. albicans.* (Control: 3% DMSO; standard: streptomycin/oxytetaracyline; ZnO NP: zinc oxide nanoparticles; Ag/ZnO NP: silver zinc oxide nanoparticles (ZnO–Ag).)

The performance of ZnO NPs and ZnO–Ag NPs as antibacterial agents against Gram positive and Gram negative pathogens, respectively, was tested using the agar well diffusion technique (Fig. 7). The result of the culture plate suggests that the ZnO–Ag NPs are more effective than ZnO NPs and comparable to the standard antibiotics. *S. typhi*, and *E. coli* displayed zone inhibition on a Gram-negative bacterial culture, as determined by measuring the inhibition zone of culture plates. Among the Gram-negative bacteria treated with ZnO–Ag NPs, *S. typhi* demonstrated the highest inhibition zone (31.6 mm), followed by *E. coli* (30.88 mm). Comparing these findings to the ZnO NP-treated zone of inhibition, certain bacteria, such as *S. typhi* and *E. coli*, have a smaller zone of inhibition (20.8 and 20.0 mm, respectively) (Table 2). Similarly, two human pathogenic Gram positive bacteria (*B. subtilis* and *S. aureus*) are similarly treated with the synthesized nanomaterial, and their effectiveness is equivalent to that of Gram negative organisms. The optimum zone of inhibition observed for *B. subtilis* (35.5 mm) and for *S. aureus* was 36.83 mm, which is also higher than that of the standard antibiotic. However, as for the above, these outcomes are higher than the ZnO NPs treated with the aforementioned Gram-positive bacteria, which are *B. subtilis* and *S. aureus*. The zones of inhibition assessed were 21.1 mm and 25.5 mm, respectively. For the control, 3% v/v DMSO has no bactericidal activity in any microorganisms tested (Table 2). Chauhan *et al.* performed a similar characterization on these Gram-negative pathogens using *Cannabis sativa* leaf extract-mediated ZnO and Ag-doped ZnO NPs, and the obtained results matched these experimental results, indicating that these nanomaterials have antimicrobial properties against the Gram-negative strains.^36^

**Table 2:** Antimicrobial sensitivity assay of ZnO NPs and ZnO–Ag NPs against tested microorganisms^a^

Test organism	Type of organism (gram stain)	Zone of inhibition (mm)			
		ZnO–Ag NPs 5 mg mL^−1^)	ZnO NPs (5 mg mL^−1^)	Standard (5 mg mL^−1^)	Control (3% DMSO)
*E. coli*	Negative	30.88 ± 1.05	20.00 ± 0.86	26.44 ± 1.01*	—
*S. typhi*	Negative	31.66 ± 1.21	20.83 ± 0.75	28.16 ± 0.75*	—
*B. subtilis*	Positive	35.50 ± 1.22	21.16 ± 0.75	28.83 ± 1.72*	—
*S. aureus*	Positive	36.83 ± 0.75	25.5 ± 0.54	33.16 ± 0.98*	—
*C. albicans*	Fungi	35.16 ± 1.72	26.33 ± 0.81	32.83 ± 1.60^#^	—
*A. niger*	Fungi	32.33 ± 0.81	23.16 ± 1.16	30.66 ± 1.50^#^	—

a All the results were expressed in mean ± SD, *streptomycin # oxytetracycline.

**Fig. 8:**
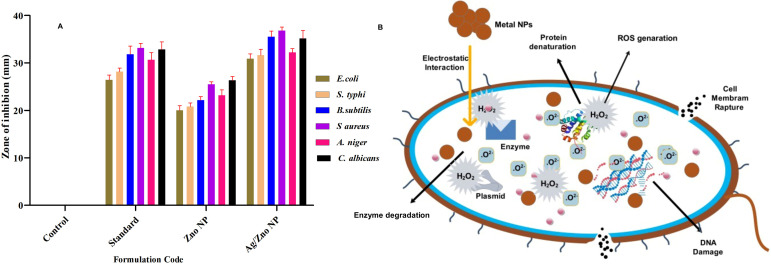
(A) Antimicrobial activity zone of inhibition of ZnO NPs and ZnO–Ag NPs against tested organisms. (B) Bactericidal mechanism of ZnO–Ag nanoparticles.

Similar to the bactericidal effect, both nanoparticles are tested against two human pathogenic fungus stains: *C. albicans* and *A. niger.* The zone of inhibition values is presented in Table 2, which indicates that ZnO–Ag NPs can inhibit the pathogens *C. albicans* (35.16 mm) and *A. niger* (32.33 mm) better than the standard antibiotic and ZnO NPs. The synthesized bimetallic ZnO–Ag NPs exhibited superior bactericidal and fungicidal activity compared to ZnO nanoparticles against all the pathogens tested in this study (Fig. 8A). In the future, there may be an alternative to chemically manufactured antibiotics that could be tested for the reversal of antimicrobial resistance.

The bactericidal activity of ZnO NPs is caused by the interaction between the metal NPs and the microorganism's membrane proteins. Numerous studies have indicated that ZnO NPs may permeate the microorganism cell wall membrane and enter the cell, forming various superoxide radicals (the production of ROS), which cause DNA damage and cell death.^55^ Similarly, the bimetallic ZnO–Ag NPs exhibited potent antibacterial and antifungal action against Gram-positive human pathogens and fungi. Owing to the presence of Ag^+^ and Zn^2+^ ions in the ZnO–Ag nanoparticle, the bactericidal and fungicidal effects were amplified.^56^ This is due to the possibility of ZnO–Ag NPs sticking to and penetrating pathogen cell walls, causing structural damage, cellular component spillage, and bacterial mortality^55^ (Fig. 8B). The increased adherence of NPs to cell membranes could be caused by their positive ion concentration, which electrostatically attracts the oppositely charged cell membranes of microorganisms. This results in morphological alterations, including cytoplasmic contraction, cell wall separation, and cell membrane rupture.^57,58^ In addition, the zinc and silver ions produced by ZnO–Ag NPs may enter microorganisms, providing the synergistic effect of preventing the multiplication or synthesis of different biological components.^59,60^ This suggests that the use of bimetallic MNPs for broad-spectrum antimicrobial applications is beneficial.

The minimum inhibitory concentrations of ZnO–Ag NP against Gram-negative organisms *S. typhi* and fungi *C. albicans* were found to be 32 μg mL^−1^ and 256 μg mL^−1^ for ZnO NPs, respectively. The MIC found for other microorganisms, such as *E. coli*, *S. aureus*, and *A. niger* was 64 μg mL^−1^ when treated with ZnO–Ag NPs, which is lower than the MIC for the same microorganisms treated with ZnO NPs (512 μg mL^−1^). “The minimum bactericidal concentration (MBC) and minimum fungicidal concentration (MFC)” were evaluated with the aforementioned microorganisms. Table 3 shows that all the microorganisms treated with the ZnO–Ag NPs had a lower MBC and MFC than the same microorganisms treated with only ZnO NPs.^21^ Determination of the MIC and MBC/MFC is a very important parameter for antibacterial or antifungal applications. Similar to this study, Saidani *et al.* also synthesized ZnO and Ag/ZnO nanoparticles (NPs) without subjecting them to post-synthesis heat treatment. Additionally, the researchers examined the MIC and MBC of their synthesized materials against Gram-negative and Gram-positive microorganisms, which are very effective against them.^61^

**Table 3:** MIC, MBC, and MFC of ZnO NPs and ZnO–Ag NPs^a^

Test organism	ZnO NPs		ZnO–Ag NPs	
	MIC	MBC	MIC	MBC
*S. typhi*	256	512	32	64
*E. coli*	512	1024	64	128
*S. aureus*	512	1024	64	128
*A. niger*	512	1024 (MFC)	64	128 (MFC)
*C. albicans*	256	512 (MFC)	32	64 (MFC)

a MIC, MBC, and MFC values were expressed in μg mL^−1^.

**Fig. 9:**
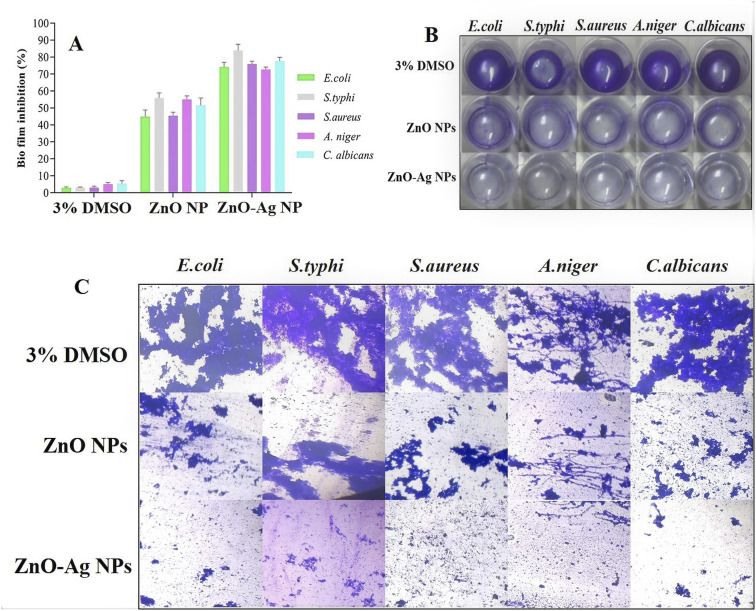
(A) The ability of ZnO–Ag nanoparticles and ZnO nanoparticles to prevent biofilm formation was evaluated. (B) The visible effect of the inhibitory action of ZnO–Ag nanoparticles and ZnO nanoparticles on biofilm formation was observed macroscopically in the tested organism. (C) The antibiofilm activity of ZnO–Ag nanoparticles and ZnO nanoparticles was visualized using light microscopy.

The production of biofilms is an essential survival strategy for microorganisms. Recent studies have indicated that people are likely to ingest biofilms, providing a highly infectious dosage of microbes. The emergence of multidrug resistance biofilms and the ineffectiveness of therapy make microbial diseases a significant worldwide concern. Because biofilms are recognized to induce resistance to various antimicrobial treatments, we examine the effects of ZnO–Ag NPs and ZnO NPs on various pathogenic biofilms.^62^ The biofilm inhibition ability of both ZnO–Ag NPs and ZnO NPs was evaluated at sub-MIC concentrations. Biofilms are 100–1000 times more antibiotic-resistant than free-living bacteria. Owing to their significant characteristics, biofilms are today one of the most critical challenges in medical science.^63^ The present investigation indicates that stable ZnO–Ag NPs suppress biofilm formation in harmful bacteria more effectively than ZnO NPs. At sub–MIC doses of ZnO–Ag NPs, it inhibited *S. typhi* and *C. albicans* the most (83.82 and 77.64%, respectively), whereas *E. coli and S. aureus* biofilms were inhibited at 74.22% and 75.93%, respectively (Fig. 9A). The ZnO NPs significantly inhibited biofilm formation against the examined microorganisms. Optical imaging has shown the antibiofilm efficacy of ZnO–Ag NPs and ZnO NPs (Fig. 9B). The ZnO–Ag NPs more significantly reduced the production of biofilms in contrast to the ZnO NPs. Furthermore, samples were examined under a light microscope to validate biofilm inhibition, and the results are shown in Fig. 9C. The control shows thick bacterial cell clumps, a characteristic of biofilm formation. However, ZnO–Ag NPs decreased the thick cell clumps more effectively than ZnO NPs. Many researchers have used ZnO NPs and ZnO–Ag NPs to inhibit biofilm forms of many bacterial species, which is consistent with our findings.^59,64^ The experiment demonstrates the ability of ZnO NPs, and bimetallic ZnO–Ag NPs to effectively suppress biofilm development by microorganisms. Khan *et al.* synthesized Ag@ZnO nanoparticles (NPs) to treat chronic wound infections with *Hibiscus sabdariffa* leaf extracts. The antimicrobial and antibiofilm properties of Ag@ZnO NPs against *S. aureus* and MRSA were investigated, and the results confirmed their significant effectiveness.^59^

**Fig. 10:**
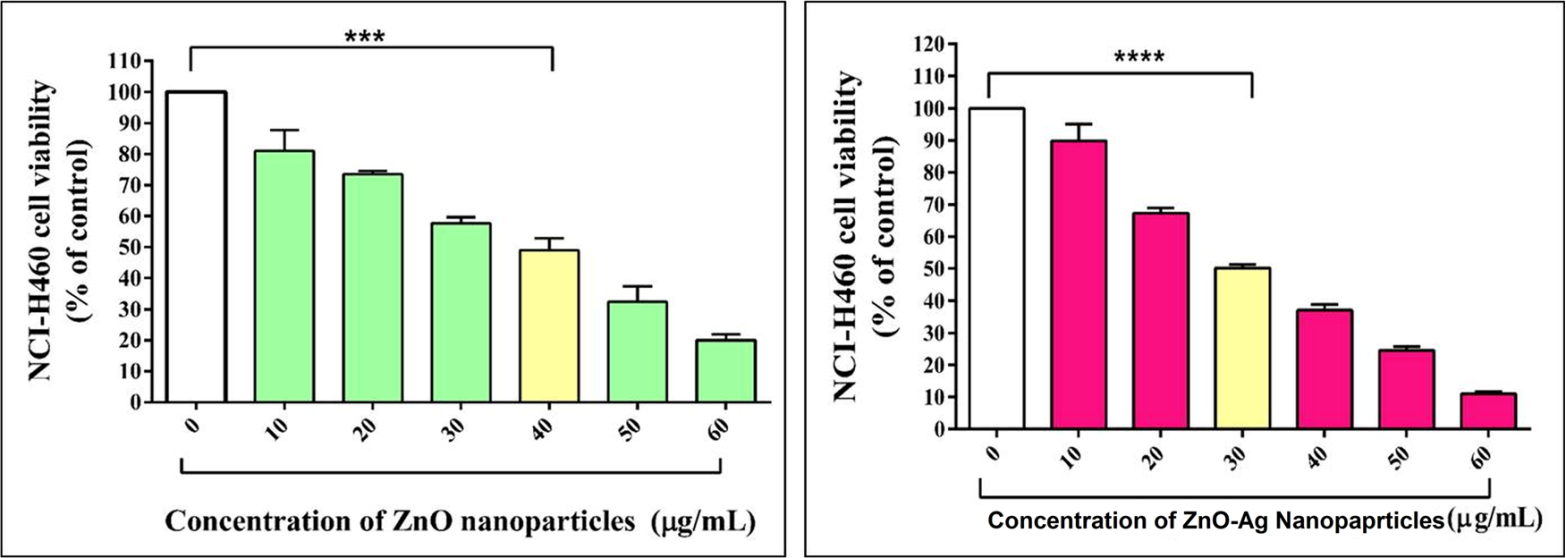
Cell line cytotoxicity analysis of ZnO NPs and ZnO–Ag NPs against the NCI-H460 cancer cell line.

The *T. dioica* seeds facilitated ZnO NPs, and ZnO–Ag NPs demonstrated considerable cytotoxicity activity toward the human lung cancer cell line NCI-H460 at varying concentrations. Cell viability depends on the activity of the cell powerhouse mitochondria. The MTT assay quantitatively assays the cytotoxicity of nanoparticles. The cells are treated for 24 h with various concentrations of ZnO NPs and ZnO–Ag NPs ranging from 10 to 60 μg mL^−1^ (Fig. 10). ZnO NPs exhibit very high toxicity against cancer cell lines. After 24 h of exposure, the ZnO NP concentration of 40 μg mL^−1^ inhibited 50% of NCI-H460 cell viability, indicating the half-maximal inhibitory concentration (IC_50_), suggesting that this concentration of the ZnO NPs significantly decreases the NCI-H460 cells in the *in vitro* MTT assay. The ZnO–Ag NPs show enhanced anticancer activity in the MTT assay. The IC_50_ value found for this nanocomposite was 30 μg mL^−1^ against the NCI-H460 human cancer cell line after exposure of 24 h, indicating that the ZnO–Ag NPs has higher toxicity towards the cancer cell line at a lower concentration than the ZnO NPs. The addition of silver to zinc oxide nanoparticles may enhance the interaction between the cell membrane and organelles, leading to faster and greater production of reactive oxygen species, rupturing of the cell membrane, cell shrinkage, and DNA damage, leading to apoptosis of NCI-H460 cells. Many studies have been conducted to prove the cytotoxicity potential of ZnO–Ag and ZnO NPs in reducing cancer progression.^65–67^ Saranya *et al.* created ZnO and Ag–ZnO NPs and evaluated them against the A549 cancer cell line, and they found that their product has cytotoxic ability over the A549 cell line.^67^ Selvan *et al.* utilized green synthesis techniques to generate Ag/ZnO nanocomposites and evaluated their anticancer efficacy on multiple cancer cell lines, including breast (MCF-7 and MDA-MB-231), colon (HCT-15), and lung (A549) cells. Using the MTT assay, the researchers found that their product had significant anticancer potential against all tested cancer cell lines tested.^66^ It is evident from these examples that ZnO–Ag NPs can destroy cancer cells. It is known that these nanoparticles trigger oxidative stress in cancer cells through the production of ROS, which damages cellular components and leads to the apoptosis of the cells. In addition, they can disrupt essential cellular processes, such as the integrity of the cell membrane and intracellular signalling networks, which are essential for cell growth and survival. In addition, ZnO–Ag NPs may target mitochondria, affecting their function and initiating apoptotic pathways in cancer cells.

**Fig. 11:**
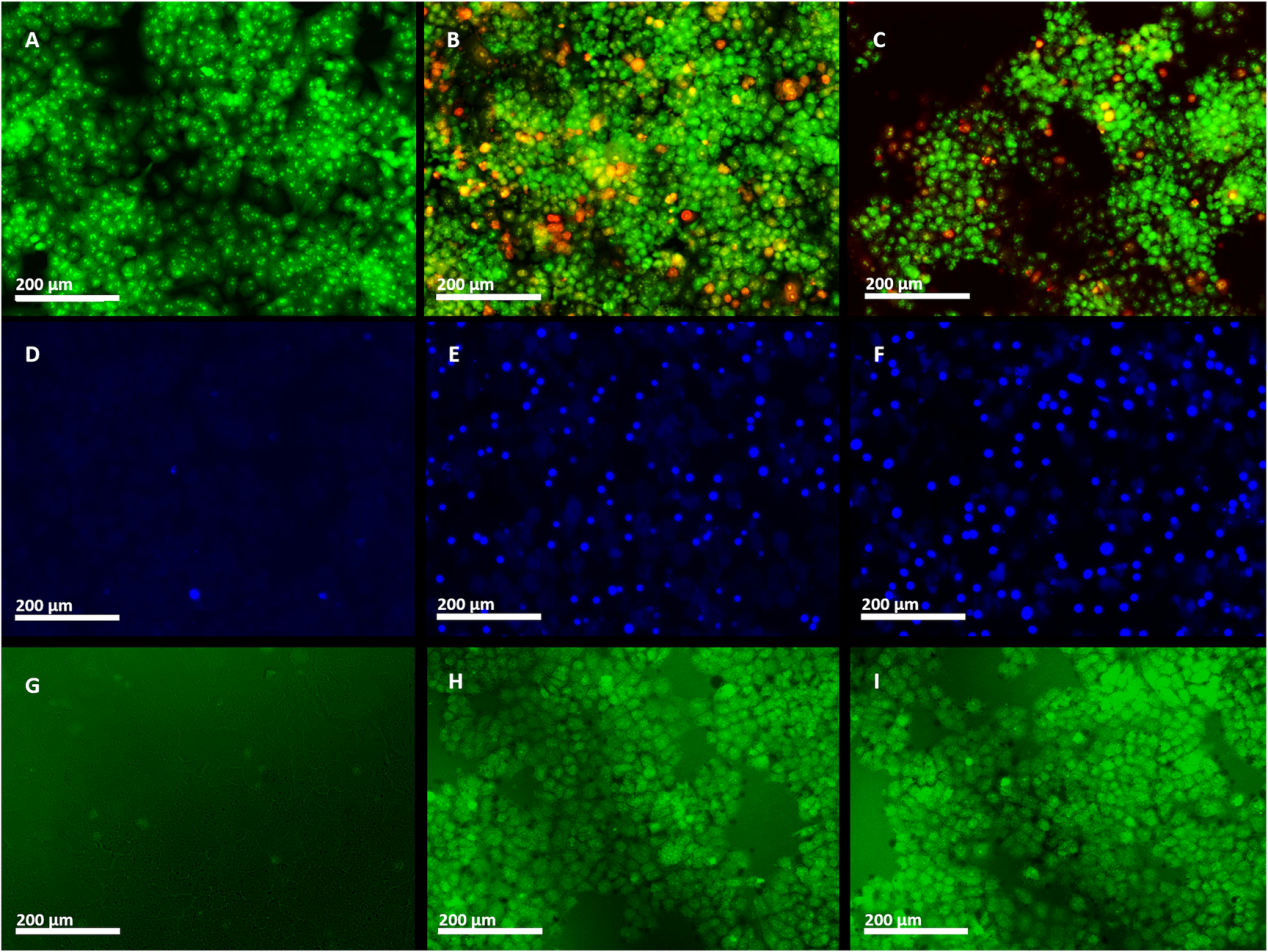
(A)–(C) AO/EtBr staining assay; (D)–(F) Hoechst staining assay; (G)–(J) DCFH-DA stain assay against NCI-H460 cell line treated with ZnO NPs and ZnO–Ag NPs. Here, (A), (D), and (G) represent the control; (B), (E), and (H) are treated with ZnO–Ag NPs; and (C), (F), and (I) represent cells treated with ZnO NPs.

The morphological and intercellular changes, including chromatin condensation and cell shrinkage of ZnO NPs, and ZnO–Ag NP treated apoptotic NCI-H460 cancer cell line, were examined by employing the AO/EtBr staining assay (Fig. 11A–C). Through the application of AO/EtBr staining and fluorescence microscopy, normal, apoptotic, and necrotic cells were observed and differentiated into four separate phases based on their fluorescence. Viable cells exhibited light green fluorescence, whereas beginning apoptotic cells exhibited brilliant green fluorescence with condensed chromosomes, late apoptotic cells exhibited orange fluorescence, and nonviable cells exhibited red fluorescence. Live cells stained with AO either exhibit green fluorescence, which indicates intact DNA, or red fluorescence, indicating DNA fragmentation. The EtBr specifically stains apoptotic cells with red fluorescence owing to fragmented DNA. Under a fluorescence microscope, this staining technique distinguishes between live cells (green) and apoptotic cells (orange/red).^65,68^ After 24 h of treatment with IC_50_ doses of the two nanoparticles, the NCI-H460 cells revealed apoptotic modifications.^69^ Both ZnO NPs and ZnO–Ag NPs enhanced the number of apoptotic cells. The nuclei and cytoplasm of the control cells were not subjected to these pathological alterations, and they fluoresced evenly green. The findings demonstrated that ZnO NPs and ZnO–Ag NPs could destroy cells through apoptosis. Similarly, Shahnaz *et al.* utilized Artocarpus heterophyllus extract to synthesize ZnO nanoparticles (NPs) and assessed their anticancer effects using an MTT assay on the HCT-116 colon cancer cell line. The study revealed that the synthesized ZnO NPs exhibited notable anticancer potential, with an IC_50_ value of 20 μg mL^−1^. Additionally, the AO/EtBr staining assay demonstrated morphological changes indicative of apoptosis in the treated cells.^83^

In this investigation, the IC_50_ concentrations of green synthesized ZnO NPs and ZnO–Ag NPs, and Hoechst (HO) dyes were used to examine the execution of apoptosis in the NCI-H460 cell line^68,70^ (Fig. 11D–F). Hoechst staining was used to examine apoptosis-induced nuclear modifications in the NCI-H460 cells. DNA binds to Hoechst, a blue fluorescent dye that easily penetrates cellular membranes. Apoptosis causes chromatin condensing and DNA fragmentation, which do not occur in living or healthy cells. As Hoechst stain binds to DNA, it produces a brilliant stain in apoptotic cells and a dull stain in normal cells, allowing for easy detection of chromatin condensation during apoptosis.^71^ To evaluate apoptosis induction, untreated and treated cancer cells were analyzed 24 h after exposure to ZnO NPs and ZnO–Ag NPs. Hoechst staining emitted faint blue fluorescence from the nuclei of the untreated control cells. In contrast, cells treated with nanoparticles exhibited apoptotic nuclei with intense blue fluorescence and condensed and fragmented nuclei, which demonstrates morphological changes in the nucleus.^68^ Ananthalakshmi *et al.* prepared a green synthesized ZnO NP and evaluated its anticancer potential on Huh7 cells.^72^ Hoechst staining reveals altered nucleus morphology and apoptotic cells exhibit bright blue fluorescence, as reported by the researchers.

The production of reactive oxygen species potentially promotes apoptosis in cells. ROS generates “hydrogen peroxide (H_2_O_2_), superoxide (O˙^2−^), and hydroxyl radical (OH˙)” that damage the cell's fundamental components, including DNA and proteins, leading to cell death.^73,74^ A fluorescent DCFH-DA stain was used to determine the degree of intracellular ROS production under a fluorescence microscope (Fig. 11G–I). There were no visible fluorescence spots on the control plate (Fig. 11G), but vivid green fluorescence spots were obtained after 24 h of treatment of NCI-H460 cells with investigational materials, which demonstrates an increase in ROS levels. Elevated ROS levels cause damage to membrane phospholipids that are attacked by free radicals, causing damage to mitochondria, DNA, and proteins. The increased ROS level in cancer cells plays a crucial role in triggering apoptosis.^73,74^ The findings indicate that these materials possess promising anticancer properties by inducing ROS generation within cancer cells.

Similarly, numerous investigations have compared the cytotoxic effects of ZnO NPs and ZnO–Ag NPs on noncancerous healthy cell lines, such as BEAS-2B, vero, and NIH/3T3 cells. Researchers have reported that similar to cancer cells, ZnO or ZnO–Ag NPs exhibit significant cytotoxicity towards normal, healthy cells. For instance, Li *et al.* evaluated nano ZnO NPs using the NIH/3T3 cell line and observed a low IC_50_ value at concentrations ranging from 5 to 15 μg mL^−1^.^75^ Similarly, Mohamed *et al.* reported lower IC_50_ values for green synthesized ZnO NPs on two healthy cell lines, Vero and clone-9, compared to the Caco-2 cancer cell line.^54^ Another study conducted by Heng *et al.* demonstrated comparable LD_50_ values for RAW-264.7 and BEAS-2B cells in cytotoxicity assessments using the WST-8 assay. Additionally, ROS generation was quantified using the DCFH-DA assay in BEAS-2B cells, revealing an increase in ROS intensity with nanoparticle concentration.^76^ Recently, Babayevska *et al.* evaluated different morphological forms of ZnO NPs against an MSU1.1 healthy cell line. Interestingly, these NPs exhibited biocompatibility towards the cervical cancer cell line (HeLa) and normal human fibroblasts (MSU1.1) at concentrations below 100 μg mL^−1^.^50^

In a different study conducted by Hashem *et al.*, the researchers examined the potential anticancer effects of biosynthesized bimetallic Ag–ZnO NPs against the vero, MCF7, and Caco2 cell lines. Their findings indicated that Ag–ZnO NPs demonstrated anticancer properties in MCF7 and Caco2 cells, with IC_50_ values of 104.9 μg mL^−1^ and 52.4 μg mL^−1^, respectively. Importantly, these concentrations were deemed safe for use, as the results of cytotoxicity testing on the Vero cell line confirmed an IC_50_ value of 155.1 μg mL^−1^.^77^ Furthermore, in another study by Khan *et al.*, similar IC_50_ values were observed for cancerous cells and 3T3 cells treated with Ag/ZnO NPs. The reported IC_50_ value in their research was 50 μg mL^−1^.^59^ The information provided strongly suggests that ZnO and Ag/ZnO nanoparticles have cytotoxic effects on cancerous and healthy human cells. This effect may be attributed to the interaction between the metal nanoparticles and the cells, resulting in the generation of reactive oxygen species and the subsequent initiation of apoptosis. In this study, it is found that the synthesized materials can also generate ROS against the NCl-H460 cell line.

**Fig. 12:**
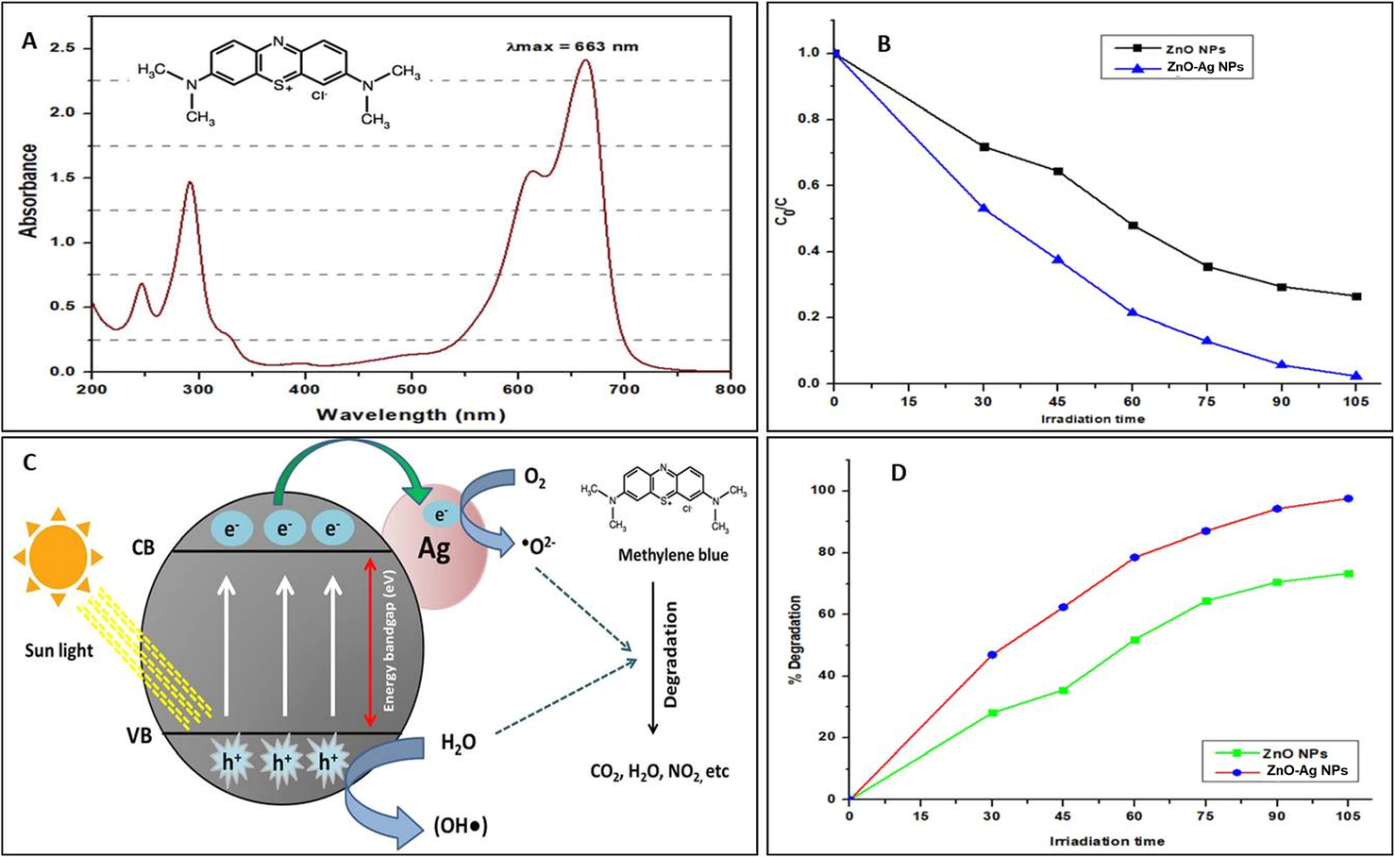
(A) The UV spectrum of methylene blue; (B) and (D) solar light treatment of dyes following exposure to ZnO or ZnO–Ag nanoparticles results in their degradation; (C) the degradation mechanism of methylene blue dyes under solar irradiation involves the quantification of dye concentration at specific time intervals (*C*_0_ – initial concentration; *C* – concentration at a time; irradiation time – min).

The results of the photocatalytic dye degradation are presented in Fig. 12. The results indicated that the degradation of MB dye was excellent when using the *T. dioica*-mediated ZnO NPs and ZnO–Ag NPs. The ultraviolet (UV) spectrophotometer was used to determine the maximum wavelength (*λ*_max_) of MB, which was found to be 663 nm. This *λ*_max_ was used to determine the absorption of the dye solution placed under solar irradiation. Fig. 12B and D show the degradation pattern of MB under solar irradiation. It is clear that nearly 50% of the MB was degraded during the first 30 min of solar irradiation of the dye solution with ZnO–Ag NPs. In contrast, only about 28% of the MB was degraded when the dye solution was treated with ZnO NPs simultaneously and under the same atmospheric conditions. After 105 min of solar irradiation with ZnO–Ag NPs, more than 90% of the MB was degraded. The graphs show that ZnO–Ag NPs perform better MB degradation (nearly 100%) than ZnO NPs alone. Several researchers have also discovered similar findings in their research, which further support the validity of our methods and findings on the photocatalytic degradation of MB dyes by ZnO–Ag NPs.^78,79^

The ability of ZnO and ZnO–Ag NPs to exhibit photocatalytic activity is due to an impressive mechanism. Essentially, ZnO acts as a photosensitizer that generates an electron–hole pair when exposed to solar radiation, which occurs when photons from sunlight interact with the surface of the material.^80^Fig. 12C depicts the photocatalytic dye degradation pathways. Many researchers claim that when a photocatalyst is exposed to sunlight, electrons from the valence band are stimulated to the conduction band, resulting in electron holes in the valence band and free electrons in the conduction band. These electron holes and free electrons engage in an oxidation–reduction reaction at the photocatalyst's surface.^80^ The electron holes combine with the surrounding moisture to form super-hydroxide radicals (OH˙), while the free electrons of the conduction band react with ambient oxygen or oxygen dissolved in the dye solution to form superoxide molecules (O˙^2−^). These superoxide molecules react with the organic dyes, destroying their chemical structure and producing CO_2_, H_2_O, and other byproducts.^29,36^ It is obvious that materials with a lower energy bandgap perform better as photocatalysts under visible light or solar energy.^81^ In our experiment, we found that the synthesized ZnO NPs and ZnO–Ag NPs have bands of 3.2 eV and 2.21 eV, respectively. From the results, we found that under the same concentration and atmospheric conditions, the ZnO–Ag NPs performed better photocatalyst activity, and nearly 100% of MB was degraded in 105 min of solar irradiation. The reason for this is that when a photon interacts with ZnO, it promotes an electron from the valence band to the conduction band, which then transfers to the silver core. This silver core acts as a reservoir of photo-excited electrons, increasing their lifetime and preventing their recombination with electron holes, which enhances the photocatalytic activity of ZnO–Ag NPs.^61^ Then, these photo-excited electrons undergo an oxidation–reduction reaction and create superoxide molecules, which degrade the MB dye more effectively than ZnO NPs.^82^ According to these findings, these greenly synthesized nanoparticles can be used to treat wastewater from various industries, such as paper, textiles, and rubber, using only solar energy.

## 4. Conclusion

*Trichosanthes dioica* seed extract was used to successfully produce ZnO and ZnO–Ag NPs using an environmentally friendly method. The seed extracts effectively reduced, capped, and stabilized the nanoparticles during synthesis. The bandgap and excitation peaks of both nanomaterials were identified by UV spectroscopy analysis. The FTIR study supported the existence of several organic functional groups. The XRD spectrum identified the crystalline nature and crystalline size of the nanoparticles. The morphological and elemental investigations performed using TEM, EDAX, and XPS prove that Ag is present on the surface of ZnO NPs. The synthesized ZnO–Ag NPs exhibit better anticancer activity than ZnO NPs against the NCl-H460 cancer cell line. These nanoparticles can produce ROS inside the cell and cause DNA fragmentation, leading to cell apoptosis, which has been identified under a fluorescence microscope. This result indicates that these materials can be used as anticancer agents. The antimicrobial properties of ZnO–Ag nanoparticles were found to be superior to those of ZnO nanoparticles and commonly used antibiotics. These inhibitory effects were also observed in the biofilm inhibition assay, in which ZnO–Ag nanoparticles demonstrated greater efficacy. Additionally, the minimum inhibitory concentration (MIC) of ZnO–Ag nanoparticles was lower than that of ZnO nanoparticles. In the photocatalytic dye degradation assay, the MB solution was degraded by 98% in 105 min using ZnO–Ag NPs under direct solar light irradiation. ZnO NPs can degrade only 70% of MB. In comparison, green synthesized ZnO–Ag NPs outperformed ZnO in every application evaluated, demonstrating its significant potential in multiple applications. The results suggest that these NPs might be employed to clean industrial wastewater by breaking down organic compounds and inhibiting the growth of dangerous microorganisms, in addition to serving as an anticancer and antibacterial agent for biomedical treatment. Future studies can investigate the biological and environmental applications of bimetallic nanocomposites, including analyzing their toxicity, developing pharmaceutical formulations, evaluating their anticancer and antimicrobial effects on *in vivo* animal models, and exploring their potential for treating wastewater from various sources. Additionally, efforts can be made to develop more eco-friendly and sustainable multipurpose nanometal composites to address the growing need for environmentally conscious and cost-effective solutions in various fields.

## 5. Future aspect

Future studies in the field of ZnO–Ag nanoparticles should focus on elucidating the underlying mechanisms behind their enhanced anticancer, antimicrobial, and photocatalytic properties. Exploring their potential in different cancer cells and pathogens, reducing their toxicity towards healthy cells, and developing biosensors for different biological molecules. Along with biological applications, photocatalytic and semiconducting efficiencies need to be optimized for various other applications. Conducting comprehensive toxicological evaluations will provide vital information regarding their safety profiles and guide the development of guidelines for their responsible use and disposal. Additionally, efforts should focus on scaling up the production of ZnO–Ag nanoparticles while maintaining the eco-friendly and sustainable nature of their synthesis. These endeavors will contribute to further advancing the potential of ZnO–Ag nanoparticles in addressing cancer, microbial infections, water pollution, and many more.

## Author contributions

The authors confirm contribution: study conception and design: Dr Abimanyu Sugumaran; study execution, data collection, draft manuscript preparation: Gouranga Dutta; analysis and interpretation of results: Dr Abimanyu Sugumaran, Gouranga Dutta, Dr Santosh kumar Chinnaiyan; correction proof & supervison: Dr Abimanyu Sugumaran & Dr Damodharan Narayanasamy. The final version of the manuscript was approved by all authors after reviewing the results.

## Conflicts of interest

The authors state that they do not have any personal relationships or competing financial interests that could have potentially influenced the work presented in this paper.

## Supplementary Material

## References

[cit1] Ferlay J., Colombet M., Soerjomataram I., Parkin D. M., Piñeros M., Znaor A., Bray F. (2021). Int. J. Cancer.

[cit2] Litwin M. S., Tan H.-J. (2017). JAMA.

[cit3] Mehrabi M. R., Soltani M., Chiani M., Raahemifar K., Farhangi A. (2023). Nanomater.

[cit4] Du F., Ma J., Gong H., Bista R., Zha P., Ren Y., Gao Y., Chen D., Ran X., Wang C. (2022). Front. Endocrinol..

[cit5] Manzoor J. and Sharma M., in Impact of Textile Dyes on Public Health and the Environment, IGI Global, 2020, pp. 162–169

[cit6] Patel H., Yadav V. K., Yadav K. K., Choudhary N., Kalasariya H., Alam M. M., Gacem A., Amanullah M., Ibrahium H. A., Park J. W., Park S., Jeon B. H. (2022). Water.

[cit7] Reddy B. L., Jatav H. S., Rajput V. D., Minkina T., Ranjan A., Harikrishnan A., Veena V. K., Chauhan A., Kumar S., Prakash A., Prasad R. (2022). J. Nanomater..

[cit8] de Boer S., González-Rodríguez J., Conde J. J., Moreira M. T. (2022). J. Water Process. Eng..

[cit9] Lellis B., Fávaro-Polonio C. Z., Pamphile J. A., Polonio J. C. (2019). Biotechnol. Res. Innov..

[cit10] Dihom H. R., Al-Shaibani M. M., Radin Mohamed R. M. S., Al-Gheethi A. A., Sharma A., Bin Khamidun M. H. (2022). J. Water Process. Eng..

[cit11] Andersson A., Harir M., Gonsior M., Hertkorn N., Schmitt-Kopplin P., Kylin H., Karlsson S., Ashiq M. J., Lavonen E., Nilsson K., Pettersson Ä., Stavklint H., Bastviken D. (2019). Environ. Sci.: Water Res. Technol..

[cit12] Afzal O., Altamimi A. S. A., Nadeem M. S., Alzarea S. I., Almalki W. H., Tariq A., Mubeen B., Murtaza B. N., Iftikhar S., Riaz N., Kazmi I. (2022). Nanomaterials.

[cit13] Dutta G., Sugumaran A. (2021). J. Drug Delivery Sci. Technol..

[cit14] Yaqoob A. A., Ahmad H., Parveen T., Ahmad A., Oves M., Ismail I. M. I., Qari H. A., Umar K., Mohamad Ibrahim M. N. (2020). Front. Chem..

[cit15] Chandrakala V., Aruna V., Angajala G. (2022). Emergent Mater..

[cit16] Saravanan A., Kumar P. S., Karishma S., Vo D.-V. N., Jeevanantham S., Yaashikaa P. R., George C. S. (2021). Chemosphere.

[cit17] CFR , FOOD and DRUGS Title 21 (CITE: 21CFR182.8991), Code Fed. Regul., 2020, vol. 3, https://www.accessdata.fda.gov/scripts/cdrh/cfdocs/cfcfr/cfrsearch.cfm?fr=182.8991

[cit18] Batra V., Kaur I., Pathania D., Sonu, Chaudhary V. (2022). Appl. Surf. Sci. Adv..

[cit19] Tehri N., Vashishth A., Gahlaut A., Hooda V. (2022). Inorg. Nano-Met. Chem..

[cit20] Burduşel A.-C., Gherasim O., Grumezescu A. M., Mogoantă L., Ficai A., Andronescu E. (2018). Nanomaterials.

[cit21] Noohpisheh Z., Amiri H., Farhadi S., Mohammadi-gholami A. (2020). Spectrochim. Acta, Part A.

[cit22] Chinnaiyan S. K., Pandiyan R., Natesan S., Chindam S., Gouti A. K., Sugumaran A. (2022). J. Drug Delivery Sci. Technol..

[cit23] Kavinkumar T., Varunkumar K., Ravikumar V., Manivannan S. (2017). J. Colloid Interface Sci..

[cit24] Samuel M. S., Ravikumar M., John A., Selvarajan E., Patel H., Chander P. S., Soundarya J., Vuppala S., Balaji R., Chandrasekar N. (2022). Catalysts.

[cit25] Ying S., Guan Z., Ofoegbu P. C., Clubb P., Rico C., He F., Hong J. (2022). Environ. Technol. Innovation.

[cit26] Álvarez-Chimal R., García-Pérez V. I., Álvarez-Pérez M. A., Tavera-Hernández R., Reyes-Carmona L., Martínez-Hernández M., Arenas-Alatorre J. Á. (2022). Arabian J. Chem..

[cit27] Kem A., Ansari M. R., Prathap P., Jayasimhadri M., Peta K. R. (2022). Phys. Scr..

[cit28] Manzoor U., Tuz Zahra F., Rafique S., Moin M. T., Mujahid M. (2015). J. Nanomater..

[cit29] Ziashahabi A., Prato M., Dang Z., Poursalehi R., Naseri N. (2019). Sci. Rep..

[cit30] Das B., Khan M. I., Jayabalan R., Behera S. K., Il Yun S., Tripathy S. K., Mishra A. (2016). Sci. Rep..

[cit31] Guo Y., Fu X., Liu R., Chu M., Tian W. (2022). J. Mater. Sci.: Mater. Electron..

[cit32] Choudhary M. K., Kataria J., Bhardwaj V. K., Sharma S. (2019). Nanoscale Adv..

[cit33] Chitradevi T., Jestin Lenus A., Victor Jaya N. (2019). Mater. Res. Express.

[cit34] Nagaraju G., Shivaraj U., Prashanth S. A., Shastri M., Yathish K. V., Anupama C., Rangappa D. (2017). Mater. Res. Bull..

[cit35] Matinise N., Fuku X. G., Kaviyarasu K., Mayedwa N., Maaza M. (2017). Appl. Surf. Sci..

[cit36] Chauhan A., Verma R., Kumari S., Sharma A., Shandilya P., Li X., Batoo K. M., Imran A., Kulshrestha S., Kumar R. (2020). Sci. Rep..

[cit37] JoVE , X-Ray Diffraction for Determining Atomic and Molecular Structure | Materials Engineering | JoVE, https://www.jove.com/v/10446/x-ray-diffraction, accessed 2 June 2023

[cit38] Carvalho P. M., Felício M. R., Santos N. C., Gonçalves S., Domingues M. M. (2018). Front. Chem..

[cit39] Nakatuka Y., Yoshida H., Fukui K., Matuzawa M. (2015). Adv. Powder Technol..

[cit40] Gordillo-Galeano A., Mora-Huertas C. E. (2021). Colloids Surf., A.

[cit41] Holišová V., Urban M., Konvičková Z., Kolenčík M., Mančík P., Slabotinský J., Kratošová G., Plachá D. (2021). Sci. Rep..

[cit42] Rakkesh R. A., Durgalakshmi D., Balakumar S. (2016). RSC Adv..

[cit43] Kadam A. N., Bhopate D. P., Kondalkar V. V., Majhi S. M., Bathula C. D., Tran A. V., Lee S. W. (2018). J. Ind. Eng. Chem..

[cit44] Xu H., Wei Z., Verpoort F., Hu J., Zhuiykov S. (2020). Nanoscale Res. Lett..

[cit45] Zare M., Namratha K., Alghamdi S., Mohammad Y. H. E., Hezam A., Zare M., Drmosh Q. A., Byrappa K., Chandrashekar B. N., Ramakrishna S., Zhang X. (2019). Sci. Rep..

[cit46] Fakhar-e-Alam M., Rahim S., Atif M., Hammad Aziz M., Imran Malick M., Zaidi S. S. Z., Suleman R., Majid A. (2014). Laser Phys. Lett..

[cit47] Primo J. de O., Horsth D. F., Correa J. de S., Das A., Bittencourt C., Umek P., Buzanich A. G., Radtke M., Yusenko K. V., Zanette C., Anaissi F. J. (2022). Nanomaterials.

[cit48] Sabry R. S., Rahmah M. I., Aziz W. J. (2020). J. Mater. Sci.: Mater. Electron..

[cit49] Talebian N., Amininezhad S. M., Doudi M. (2013). J. Photochem. Photobiol., B.

[cit50] Babayevska N., Przysiecka Ł., Iatsunskyi I., Nowaczyk G., Jarek M., Janiszewska E., Jurga S. (2022). Sci. Rep..

[cit51] López-Miranda J. L., España Sánchez B. L., Esparza R., Estévez M. (2022). Mater. Chem. Phys..

[cit52] Onyszko M., Zywicka A., Wenelska K., Mijowska E. (2022). Part. Part. Syst. Charact..

[cit53] González S. C. E., Bolaina-Lorenzo E., Pérez-Trujillo J. J., Puente-Urbina B. A., Rodríguez-Fernández O., Fonseca-García A., Betancourt-Galindo R. (2021). 3 Biotech.

[cit54] Mohamed A. A., Fouda A., Abdel-Rahman M. A., Hassan S. E.-D., El-Gamal M. S., Salem S. S., Shaheen T. I. (2019). Biocatal. Agric. Biotechnol..

[cit55] Franco D., Calabrese G., Guglielmino S. P. P., Conoci S. (2022). Microorganisms.

[cit56] Yang H., yu Ren Y., Wang T., Wang C. (2016). Results Phys..

[cit57] Wu Y., Yang Y., Zhang Z., Wang Z., Zhao Y., Sun L. (2018). Adv. Powder Technol..

[cit58] Parmar S., Kaur H., Singh J., Matharu A. S., Ramakrishna S., Bechelany M. (2022). Nanomaterials.

[cit59] Khan M. I., Paul P., Behera S. K., Jena B., Tripathy S. K., Stålsby Lundborg C., Mishra A. (2021). Chem. Eng. J..

[cit60] Slavin Y. N., Asnis J., Häfeli U. O., Bach H. (2017). J. Nanobiotechnol..

[cit61] Saidani M. A., Fkiri A., Smiri L. S. (2019). J. Inorg. Organomet. Polym. Mater..

[cit62] Hall C. W., Mah T.-F. (2017). FEMS Microbiol. Rev..

[cit63] Sharma D., Misba L., Khan A. U. (2019). Antimicrob. Resist. Infect. Control.

[cit64] Ishwarya R., Vaseeharan B., Kalyani S., Banumathi B., Govindarajan M., Alharbi N. S., Kadaikunnan S., Al-anbr M. N., Khaled J. M., Benelli G. (2018). J. Photochem. Photobiol., B.

[cit65] Rajendran R., Mani A. (2020). J. Saudi Chem. Soc..

[cit66] Arumai Selvan D. S., Keerthi M., Murugesan S., Shobana S., Lakshmi B., Veena V., Rahiman A. K. (2021). Mater. Chem. Phys..

[cit67] Saranya A., Murad A., Thamer A., Priyadharsan A., Maheshwaran P. (2021). ChemistrySelect.

[cit68] Wu H., Zhang J. (2018). Saudi Pharm. J..

[cit69] Venugopal K., Rather H. A., Rajagopal K., Shanthi M. P., Sheriff K., Illiyas M., Rather R. A., Manikandan E., Uvarajan S., Bhaskar M., Maaza M. (2017). J. Photochem. Photobiol., B.

[cit70] Xu X. M., Zhang Y., Qu D., Liu H. B., Gu X., Jiao G. Y., Zhao L. (2013). Exp. Ther. Med..

[cit71] Bastian A. M., Yogesh T. L., Kumaraswamy K. L. (2013). Indian J. Cancer.

[cit72] Ananthalakshmi R., Rathinam S. R. X. R., Sadiq A. M. (2021). J. Inorg. Organomet. Polym. Mater..

[cit73] Perillo B., Di Donato M., Pezone A., Di Zazzo E., Giovannelli P., Galasso G., Castoria G., Migliaccio A. (2020). Exp. Mol. Med..

[cit74] Aggarwal V., Tuli H. S., Varol A., Thakral F., Yerer M. B., Sak K., Varol M., Jain A., Khan M. A., Sethi G. (2019). Biomolecules.

[cit75] Li S., Song W., Gao M. (2013). Procedia Environ. Sci..

[cit76] Heng B. C., Zhao X., Tan E. C., Khamis N., Assodani A., Xiong S., Ruedl C., Ng K. W., Loo J. S. C. (2011). Arch. Toxicol..

[cit77] Hashem A. H., El-Sayyad G. S. (2023). Biomass Convers. Biorefin..

[cit78] Singh S. (2022). Mater. Today Commun..

[cit79] Sharwani A. A., Narayanan K. B., Khan M. E., Han S. S. (2022). Sci. Rep..

[cit80] Ong C. B., Ng L. Y., Mohammad A. W. (2018). Renewable Sustainable Energy Rev..

[cit81] Ansari S. A., Cho M. H. (2016). Sci. Rep..

[cit82] Khan M. S., Dhavan P. P., Jadhav B. L., Shimpi N. G. (2020). ChemistrySelect.

[cit83] Shahnaz M., Danish M., Ismail M. H. B., Ansari M. T., Ibrahim M. N. M. (2019). Sustainable Chem. Pharm..

